# Ethnopharmacology of Fruit Plants: A Literature Review on the Toxicological, Phytochemical, Cultural Aspects, and a Mechanistic Approach to the Pharmacological Effects of Four Widely Used Species

**DOI:** 10.3390/molecules25173879

**Published:** 2020-08-26

**Authors:** Aline T. de Carvalho, Marina M. Paes, Mila S. Cunha, Gustavo C. Brandão, Ana M. Mapeli, Vanessa C. Rescia, Silvia A. Oesterreich, Gustavo R. Villas-Boas

**Affiliations:** 1 Research Group on Development of Pharmaceutical Products (P&DProFar), Center for Biological and Health Sciences, Federal University of Western Bahia, Rua Bertioga, 892, Morada Nobre II, Barreiras-BA CEP 47810-059, Brazil; alineteixeiraufob@gmail.com (A.T.d.C.); marinameirelles@ymail.com (M.M.P.); milacunha035@gmail.com (M.S.C.); vanessa.rescia@ufob.edu.br (V.C.R.); 2 Physical Education Course, Center for Health Studies and Research (NEPSAU), Univel University Center, Cascavel-PR, Av. Tito Muffato, 2317, Santa Cruz, Cascavel-PR CEP 85806-080, Brazil; gustavochavesbrandao@gmail.com; 3 Research Group on Biomolecules and Catalyze, Center for Biological and Health Sciences, Federal University of Western Bahia, Rua Bertioga, 892, Morada Nobre II, Barreiras-BA CEP 47810-059, Brazil; mmapeli@ufob.edu.br; 4 Faculty of Health Sciences, Federal University of Grande Dourados, Dourados, Rodovia Dourados, Itahum Km 12, Cidade Universitaria, Caixa. postal 364, Dourados-MS CEP 79804-970, Brazil; silviaoesterreich@gmail.com

**Keywords:** plant secondary metabolites, natural compounds, biological activity, phytochemistry, pharmacological activity, plant side effects, *Talisia esculenta*, *Brosimum gaudichaudii*, *Genipa americana*, *Bromelia antiacantha*

## Abstract

Fruit plants have been widely used by the population as a source of food, income and in the treatment of various diseases due to their nutritional and pharmacological properties. The aim of this study was to review information from the most current research about the phytochemical composition, biological and toxicological properties of four fruit species widely used by the world population in order to support the safe medicinal use of these species and encourage further studies on their therapeutic properties. The reviewed species are: *Talisia esculenta, Brosimum gaudichaudii, Genipa americana,* and *Bromelia antiacantha*. The review presents the botanical description of these species, their geographical distribution, forms of use in popular medicine, phytochemical studies and molecules isolated from different plant organs. The description of the pharmacological mechanism of action of secondary metabolites isolated from these species was detailed and toxicity studies related to them were reviewed. The present study demonstrates the significant concentration of phenolic compounds in these species and their anti-inflammatory, anti-tumor, photosensitizing properties, among others. Such species provide important molecules with pharmacological activity that serve as raw materials for the development of new drugs, making further studies necessary to elucidate mechanisms of action not yet understood and prove the safety for use in humans.

## 1. Introduction

The Cerrado is one of the most important and extensive Brazilian biomes, with a great richness of plant species that are used as food and therapeutic agents, highlighting their medicinal, sociocultural and nutritional importance, which make them attractive for research and commercialization [1].

The Brazilian population has great range of cultural knowledge about native plants, which are used in the treatment of diseases. These medicinal plants present molecules with pharmacological properties which expand the possibilities for the development of drugs and/or nutraceuticals. However, despite the great Brazilian biodiversity in species considered medicinal, research is still incipient, requiring active investigations on the phytochemical constituents present in these plants, as well as their pharmacological or nutritional properties [2].

Climatic factors directly affect the production of fruits and phytochemical constituents by plants, since water availability in the Cerrado is reduced over a period that can vary from two to five months. Some species adapt to reduced water availability in soil and increased temperature, being called sclerophyll plants. These plants make up the Cerrado vegetation, which is characterized by the presence of shrubs, grasses and trees with deep roots to facilitate water absorption; in the dry season, some species lose their leaves in an attempt to save water [3].

Given these environmental conditions to which Cerrado plants are exposed, another way to mitigate the damage caused by climate changes is the production of bioactive molecules that act in the defense of the plant against harmful agents. These compounds are alternative sources for the formulation of new products, not only in the pharmaceutical industry, but also in the food industry. In addition to climatic conditions associated with different geographic regions, factors such as cultivation, harvest time and growth stage of the collected plant can also change the concentration of these compounds [4].

Some Cerrado fruit species are used as functional foods, with fruits and seeds being the most used parts. The therapeutic and nutritional properties associated with these foods are constantly investigated through scientific studies, which highlight the high concentration of phenolic compounds found in these species, normally produced in response to water scarcity, intense exposure to solar radiation, attack of herbivores and infections by fungi, which are conditions common to Cerrado plants. The sale of parts of these plants for fresh consumption or therapeutic purposes has great prominence in the economy as source of livelihood for workers in regions covered by this biome [5].

Studies that have assessed the biological activity, toxicity and phytochemical composition of plants native to the Cerrado can contribute to ensuring the effectiveness and safety in the use of such species, favoring the healthy consumption of fruits and by-products, also encouraging further studies on the therapeutic properties of substances isolated from these plants, which are of great importance for popular medicine, nutrition and income in various regions of the world. In this sense, the aim of this study was to review the current literature to gather detailed and accurate information about the phytochemical, pharmacological and toxicological aspects of the following fruit plants: *Talisia esculenta, Brosimum gaudichaudii, Genipa americana, Bromelia antiacantha*.

## 2. Fruit Plants from the Brazilian Cerrado

Cerrado is considered the second largest Brazilian biome, only behind the Amazon, accounting for around 23% of the national territory and extending into 11 of the country’s states. With abundant flora and fauna diversity, this biome has been annually targeted by deforestation caused by the expansion of agribusiness, livestock and urbanization, which already occupy 50% of the biome’s extension [6].

The multiplicity of Cerrado plant species is superior to that found in other regions of the world, with shrub, liana and herbaceous plants, and the registered number reaches 12,669 species, of which, some stand out for their relevant pharmacological and nutritional properties, which play an important role in the commercial activities of regions where they are found. In addition, fruits of these plants also contribute to the promotion and development of family farming, generating income for communities through the preparation of sweets, ice cream, flavorings for alcoholic distillates and other by-products [7,8,9].

Fruits produced by Cerrado species are known not only for their flavor and aroma, which are generally striking, but also for their high concentrations of carotenoids, phenolic compounds, vitamins and minerals, whose antioxidant power is desired in the food and pharmaceutical industry. The identification and quantification of these components allow the assessment of their nutritional value and, consequently, the production and commercialization of by-products with guaranteed quality [10].

However, for the study of properties attributed to the phytochemical constituents found in fruits of these species, the correct identification of species must be performed by a botanist. Such species have their classification based on botanical nomenclatures, followed by norms created by an international commission of scientists, which are described in the International Botanical Nomenclature Code, which aims to guarantee the universality of names given to taxa. In this code, plants are categorized into Phylum, Class, Order, Family, Genus and species [11,12].

## 3. Sapindaceae Family

The Sapindaceae family belongs to the order of Sapindales angiosperm plants, has 141 genera and approximately 2000 identified species, 28 genera and about 418 species being native to Brazil. It is predominantly found in tropical and subtropical climates, with rare occurrence of some genera in countries with temperate climate [13]. This family is morphologically characterized by the presence of shrubs, lianas with tendrils and trees, whose leaves can be alternate or opposite, composed, trifoliate, unifoliated, pinnate or webbed, with present or absent stipules and unisexual and monoic flowers [14]. In addition, this plant family has species with edible fruits with industrial potential, for example, *Litchichinensis* Sonn. (lychee), *Melicoccus bijugatus* Jacq. (mamoncillo or Spanish lime), *Nephelium lappaceum* L. (rambutão) and *Talisia esculenta* (A. St. Hil) Radlk. (pitomba). Other species have known medicinal and ichthyotoxic properties, such as species of the genera *Paullinia* L. and *Serjania* Mill., and there are those that can be simply ornamental, such as *Paullinia pinnata* L. and *P. elegans* (cipó-timbó). The flowers of these species are usually tetrameric or pentameric, with an extra-stamen nectary, that is, located between the androceu and the perianth. Fruits can be dehiscent or indehiscent, ranging from berries and capsules to schizocarps [15,16,17].

### 3.1. Genus Talisia and Species Talisia esculenta

The genus *Talisia* was first described by Aublet in 1775. Soon after, in 1778, following up on Aublet’s findings, Rodlkofer carried out several studies on this genus. About 10 species of the genus *Talisia* have nutritional properties, including *Talisia esculenta* (popularly known as “pitombeira”, although the name is also used for other species of the same genus, such as *T. acutifolia* Raldk, *T. cerasina* (Benth). Radlk. and *T. cupularis* Radlk., all from the Amazon [14]).

The species has characteristics that help it adapt to areas along the margins of water courses, such as rapid growth and large seed production, which are dispersed with high water content, that is, recalcitrant seeds, which should be sown quickly, as they are only viable for a short time in the environment [18].

Fruits produced by this species are consumed by humans and birds, with economic importance attributed to their nutritional properties and characteristic flavor, desired in regional cuisine, being used in the manufacture of pulps, jams, sweets, and jellies. In addition, the wood derived from the trunks is used in the manufacture of furniture and decorative objects, while leaves and seeds have been investigated in several studies due to their reported therapeutic properties based on their popular use [19].

#### 3.1.1. Geographic Distribution and Popular Use

*T. esculenta* although native to Brazil, has a cosmopolitan distribution and occurs in several other countries such as Bolivia, Paraguay, Colombia, Ecuador, Peru and Argentina, where the climate is favorable for its development [20]. It can be found in the native and wild state or cultivated and despite being a tropical climate plant, it adapts well to subtropical areas, with preference for alluvial soils in valley bottoms. Its flowering occurs from August to October and the fruit maturation occurs between January and March, which may vary according to the region in which the species is found [21,22].

The use of *T. esculenta* by the population is mainly for food and medicinal purposes. The fruit is commonly used for fresh consumption or in the form of by-products. The other plant parts are associated with therapeutic purposes, such as leaves, which are popularly used for back pain and rheumatism, seeds for diarrhea, dehydration and as astringents and barks for kidney problems [9,23].

The therapeutic use of teas made from the leaves can vary according to the region. An example was reported in an ethnobotanical study of species with medicinal use, which describes the use of *T. esculenta* leaves tea as antihypertensive, a property little mentioned by the inhabitants of other regions of the country [24]. Regarding the form of preparation, tea is usually obtained from freshly harvested or dried plant parts produced through the method of infusion or decoction, using leaves to prepare tea by infusion, while seeds and bark are used to prepare tea by decoction. Some studies point out a concern in relation to the hygiene of plant parts to be used and the way they are dried and stored, which can favor contamination or proliferation of deteriorating microorganisms [25,26].

In the preparation of teas using the infusion method, the popularly used solvent is boiling water, in which the vegetable is immersed for about 30 min. After this time, the tea is leached and cooled until it reaches an ideal temperature for consumption. Unlike infusion, in decoction, which is the most widely used form of tea preparation, the vegetable is in direct contact with water throughout the process, from heating to boiling; then the tea is leached and cooled to be consumed. A negative consequence associated with these methods is the degradation of some thermolabile compounds that do not resist exposure to intense heat [27].

#### 3.1.2. Botanical Aspects

*T. esculenta* is an arboreal fruit plant of some 6–12 m in height, with terrestrial roots and aerial and erect cylindrical stems of dark and lenticelous in color; leaves are composite and alternate, with 2 to 4 pairs of leaflets, with petioles of 3–10 cm in length and a petiole of 1–5 mm and simple non-glandular trichomes on their surface. In addition, as to morphology, leaves are classified in oblong shape, acuminated apex, rounded or obtuse base and venation is of peninerval type [20,28].

Inflorescences are composed, of the thyrsus type, that is, forming racemes of crests. Flowers are white, aromatic, diclamid, with pedicels up to 4 mm in length, gamosepal, with five elliptical sepals and dialipetal, with five petals. Classification regarding sexual characteristics is not well defined, with monoclinous flowers, that is, hermaphrodites, or diclinous, which are unisexual male or female. They present about eight filiform and hairy stamens, oblong and apiculate anthers, trifid stigmas and ovoid ovary, tricarpellar and trilocular [14,20].

Fruit production occurs annually, about ten years after planting, and it is possible to harvest ten to twenty fruits in each raceme. Fruits are generally monospermic, globose, fleshy, drupe type and when ripe they have approximately 2.5 cm in diameter and the color of the epicarp changes from green to brown. The pulp of the ripe fruit has bittersweet flavor and its color varies from white to transparent [29].

Seeds are elongated, reddish in color immediately after harvest and dark after drying. They are surrounded by a pinkish-white aryl, which must be removed before planting, as it can harm germination. Regarding seed viability, it is about 15 days in the environment, but if stored in polyethylene package with 50% relative humidity under refrigeration (approx. 18 °C), they can remain viable for up to 25 days. Seed dispersion enables species maintenance; however, it is still not clear which agent is responsible for dissemination, since the fruits attract several animals [18,30,31].

#### 3.1.3. Phytochemical Aspects

Table 1 presents a summary of phytochemical studies carried out with different *T. esculenta* organs, as well as the structures of isolated substances.

Phytochemical investigations include the in-depth study of the target species, as well as extractive and separation methods, purification and structural determination of isolated chemical constituents [37]. Cerrado species are known to have significant amount of phenolic compounds in their composition, which are bioactive substances widely distributed in nature and derive from two biosynthetic routes, that of shikimic acid and that of acetyl-CoA, being divided into two large groups, phenolic acids and flavonoids, commonly found in fruits and vegetables [38].

To date, there is scarcity of studies on the bioactive compounds present in *T. esculenta*, mainly for roots and stems. [39] determined the centesimal composition of *T. esculenta* fruits. The average values found after triplicate analysis, were: 56.35 kcal/100 g of energy value, 83.16 g/100g of moisture, 1.15 g/100 g of proteins, 0.19 g/100 of lipids, 12.51 g/100 g of carbohydrates, 2.40 g/100 g dietary fiber and 0.61 g/100 g of fixed mineral residue. In addition, the analysis revealed 26.7 mg/100 g of calcium, 0.84 mg/100 g of zinc and 0.60 mg/100 g of iron, 0.0 mg/100 g of copper and 10.8 mg/100 g of magnesium. The phosphorus concentration was considered insignificant [40].

In a study on fruits and seeds in which extraction was performed using the technique of maceration in acetone and methanol, quinic (**9**), gallic (**5**) and *p*-coumaric (**8**) acids, in addition to epicatechin (**4**) and catechin (**3**), were identified in the ketone extract of the pulp, the latter being also found in the pulp methanolic extract. In the ketone extract of the fruit bark, naringenin (**7**), catechin (**3**) and epicatechin (**4**) were detected, not identified in the methanolic extract of the fruit bark. For seeds, epicatechin (**4**), catechin (**3**), naringenin (**7**) and luteolin (**6**) were found in the ketone extract (**6**) and in the methanolic naringenin extract (**7**) and luteolin (**6**) [33].

Other phytochemical findings of this species were analyzed using 5:95 hydroalcoholic extract (*v/v*, water, ethanol) from fruits, followed by evaporation and collection of the lipophilic fraction, which was homogenized in hexane. High mirecetin (**1**) and quercetin (**2**) concentrations were determined, approximately 89.90 mg/100 g and 30.20 mg/100 g, respectively, which are associated with possible antioxidant and antiproliferative properties [36].

Furthermore, the analysis of the methanolic extract of the pulp of *T. esculenta* fruits showed the presence of flavonoids and phenolic acids. The following 12 phenolic compounds were found: gallic acid (**5**), chlorogenic acid (**13**), catechin (**3**), epicatechin (**4**), caffeic acid (**12**), serum acid (**16**), *p*-coumaric acid (**8**), rutin (**10**), ferulic acid (**15**), quercetin (**2**), eriodicthiol (**14**) and acacetin (**11**); and the cyclitol quinic acid (**9**). This study identified 27 aromatic compounds, including esters, alcohols, aldehydes, hydrocarbons, fatty acids and terpenoids such as monoterpenolinalol (**18**) and the sesquiterpene β-bisabolene (**17**) [35].

In this sense, in order to investigate the phytochemical composition of *T. esculenta* leaves and stem, hydroalcoholic extract was produced, which, after analysis and characterization, allowed identifying derivatives of flavonoids, benzoic and cinnamic acids, fragments of aglycones, quercetin (**2**) and *dicaffeoylquinic* acid (**24**), which compounds being given the ability to influence renal hemodynamics and induce diuretic response in normotensive and spontaneously hypertensive Wistar rats [35].

Junior (2019) analyzed the hydroalcoholic extract of *T. esculenta* leaves and identified some compounds such as quinic acid (**9**), caffeic acid (**12**), gallic acid (**5**), which are classified as phenolic acids, in addition to flavonoids such as catechin (**3**), rutin (**10**), acacetin (**11**) and quercetin (**2**), which are associated with antioxidant and anti-inflammatory properties [36].

#### 3.1.4. Pharmacological Studies

Flavonoids, phytochemicals present in different *T. esculenta* parts have pharmacological properties that are the focus of several studies, in which, among others, the anti-inflammatory, antioxidant and anti-tumor properties associated with these compounds are described. A study analyzed the anti-inflammatory activity of some flavonoids with a focus on the possible regulatory role in the activation of NLRP3 inflammasome. The mechanism proposed for inhibiting activation by flavonoids was based on the regulation of the expression of inflammasome components such as the amino-terminal pyrin domain (PYD), which interacts with the ASC pyrin domain (caspase recruitment domain) to initiate the assembly of the inflammasome; the central nucleotide binding and oligomerization domain (NACHT), which has the ATPase activity necessary for NLRP3 oligomerization after activation; and C-terminal leucine-rich repeat (LRR) domain, whose function has not yet been identified. These changes can prevent its assembly and lead to inhibition of caspase-1 activation and, consequently, maturation and secretion of pro-inflammatory cytokines [41,42].

Luteolin (**6**), a flavonoid found in *T. esculenta* seeds, has anti-inflammatory activity attributed to its ability to reduce the generation of reactive oxygen species (ROS) and inhibit the activation of NLRP3 inflammasome. Previous studies have described some Pattern-Recognition Receptors (PRR) located in the cytoplasm of cells, such as dendritic cells and macrophages, which are also involved in the induction of inflammatory responses [43]. Among these receivers, some belong to the family of NOD-like receptors (NRLs). NLRs are a large family of intracellular PRRs with similar structure [44,45]. Among the various types of NLRs, NLRP3 is recognized for responding to various stimuli, being responsible for the inflammasome activation (NLRP3 inflammasome), involved in the recruitment and activation of caspase-1 (pro-caspase 1) in association with the ASC adapter protein [46]. The role of activated caspase-1 is crucial for the conversion of pro-interleukin 1 beta (pro-IL-1β) and pro-interleukin 18 (pro-IL-18) into their mature and biologically active forms [47]. Thus, luteolin (**6**) is able to reduce the expression of interleukin-1 beta (IL-1β), a cytokine with a primary role in the inflammatory response, and interleukin-8 (IL-8), a chemokine that stimulates migration of immune cells. Another anti-inflammatory flavonoid found in *T. esculenta* seeds is rutin (**10**), which promotes the inhibition of the NLRP3 inflammasome activation through the negative regulation of the NLRP3, ASC and caspase-1 expression and reduced production of IL-1β and interleukin-18 (IL-18), which is also known as an interferon-γ inducing factor (IFN-γ) [41,48].

A previous study revealed the immunoregulatory properties of naringenin (**7**), a flavonoid also found in *T. esculenta* seeds. This compound promotes the inhibition of nuclear transcription factor kappa B (NF-κB), mitogen-activated protein kinase (MAPK) and reduces the production of tumor necrosis factor alpha (TNF-α) and interleukin-6 (IL-6), a pro-inflammatory cytokine [49].

**Figure 1:**
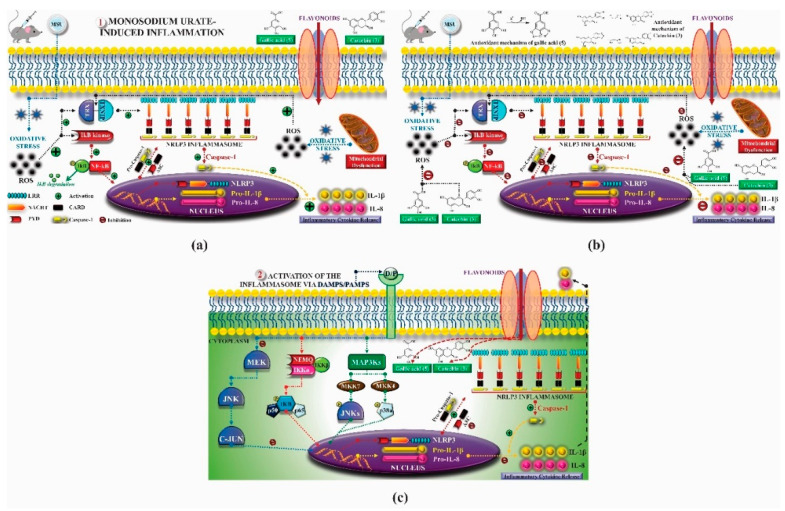
Molecular mechanism of flavonoids in inflammasome regulation: (**a**) Studies suggest that oxidative stress is an important mediator of monosodiumurate (MSU) induced inflammation [51]. The formation of reactive oxygen species (ROS) induces nuclear translocation of Nuclear Factor-kappa B (NF-kB) via phosphorylation by IkB kinase, which binds to target DNA that regulates Pro-IL-1β and Pro-IL-8 gene expression. In addition, ROS dissociates the thioredoxin (TRX) and thioredoxin interaction protein (TXNIP) conjugation [52], and released TXNIP further recruits and binds to NLRP3 inflammasome, leading to the release of IL-1β [53] and IL-8. NLRP3 inflammasome consists of NLRP3, caspase recruitment domain (ASC), and pro-caspase-1. Mitochondrial ROS (MtROS) is also associated with NLRP3 inflammasome activation [53]. In the process of NLRP3 inflammasome activation, activated caspase-1 transforms pro-IL-1b and pro-IL-18 into mature IL-1b and IL-18, resulting in the release of inflammatory cytokines; (**b**) Flavonoid uptake occurs either via passive diffusion through the cell membrane, or through membrane bound transport proteins. Cut circles indicate different points of flavonoid action, inhibiting the process of inflammasome formation with subsequent inhibition of inflammatory events [54]. Phenolic compounds block the inflammatory process by inhibiting ROS formation, thereby reducing the formation of pro-inflammatory cytokines. The nature and position of substituents in relation to the hydroxyl group affect the activity of polyphenols. The easily ionizable carboxylic group contributes to the efficient hydrogen donation tendency of phenolic acids [55]. Gallic acid has high antioxidant activity rate. This is due to a beneficial influence of carboxylate on the antioxidant activity of phenolic acids [56]. The tricyclic structure of flavonoids, such as catechin, determines their antioxidant effect. Phenolic quinoid tautomerism and the localization of electrons over the aromatic system eliminate reactive oxygen species. These aromatic rings directly neutralize free radicals and increase antioxidant defense [57]; (**c**) DAMPs/PAMPs bind to their receptor on the cell membrane and activate a signaling cascade. As a consequence, activation and formation of NRLP3 inflammasome occur, where the formation of active caspase-1 catalyzes the cleavage and secretion of mature IL-1β and IL-18, leading to propagate inflammation [54]. ASC, caspase recruitment domain; C-JUN/JNK, c-Jun N-terminal Kinase; CARD, caspase recruitment domain; DAMPs, damage-associated molecular patterns; IκB, inhibitor of κB; IKKα, IkBkinase α; IKKβ, IkBkinase β; IL-1β, Interleukin 1-beta; IL-8, Interleukin 8; LRR, leucine-rich repeats; MAP3Ks, mitogen-activated protein 3 kinases; MEK, mitogen-activated protein kinase; MKK4, mitogen-activated protein kinasekinase 4; MKK7, mitogen-activated protein kinase kinase 7; MtROS, Mitochondrial ROS; MSU, monosodiumurate; NACHT, central nucleotide-binding and oligomerization domain; NEMO, NF-kappa-B essential modulator; NF-ΚB, Nuclear Factor-kappa B; p38a, p38 kinase α; p50, NF-ΚB, Nuclear Factor-kappa B 1 (NF-ΚB1); p65, RelA; PAMPs, pathogen-associated molecular patterns; PYD, pyrin domain; ROS, reactive oxygen species; TXNIP, thioredoxin interaction protein; TRX, thioredoxin; TXNIP, thioredoxin interaction protein.

Jhang et. al. [50] analyzed the therapeutic potential of catechin (**3**) and gallic acid (**5**), both found in *T. esculenta* fruits and possible anti-inflammatory properties were found. After subcutaneous injection in mice, whose inflammation was stimulated by the administration of monosodium urate (MSU), significant reduction in the production and secretion of IL-1β and IL-6 was observed. The secretion of IL-1β was modulated through two pathways that include the NF-κB pathway, which provides pro-IL-1β and the NLRP3 inflammasome pathway, which promotes the release of IL-1β from pro-IL-1β. The study demonstrated that catechin (**3**) and gallic acid (**5**) have potent activity to eliminate superoxide anions, consequently inhibiting MSU-induced IL-1β secretion and NLRP3 inflammasome activation. Catechin (**3**) also regulated the oxidative stress status in mitochondria through positive regulation of thioredoxin (TRX) and deglycase protein DJ-1 (DJ-1) and these effects prevented mitochondrial damage caused by MSU attack. These results suggest that catechin intake has potential to prevent acute gout attacks. In addition, researchers have demonstrated the role of these compounds on the intracellular calcium concentration, which is high during the inflammatory process, with significant reduction in calcium concentration by catechin (**3**), but not by gallic acid (**5**) (Figure 1a,b).

For the inflammasome activation, danger signals such as Damage-Associated Molecular Patterns (DAMPs) or Pathogen-Associated Molecular Patterns (PAMPs) bind to PRRs on the cell membrane and trigger the activation of a signaling cascade, which includes the activation of NF-κB, MAPK and MAPK activating protein kinase (MEK), leading to activation and assembly of the inflammasome, a protein complex where active caspase-1 catalyzes the cleavage and secretion of IL-1β and IL-18, important pro- inflammatory signaling gents. In this context, flavonoids cross the membrane through passive or facilitated diffusion using membrane-bound transport proteins. In the cell, different flavonoids act in the same pathway or in different pathways, blocking the formation of inflammasome and consequently inhibiting the inflammatory process (Figure 1c) [54].

The flavonoid quercetin (**2**), found in *T. esculenta* fruits, has anti-dyslipidemic activity, which is also associated with inflammation, since the accumulation of lipids is a factor that contributes to the inflammatory response and inflammasome formation. Quercetin (**2**) acts by suppressing the NLRP3 expression, inhibiting caspase-1 and the production of IL-1β, also reducing the levels of lipids, more specifically triacylglycerols [58].

**Figure 2:**
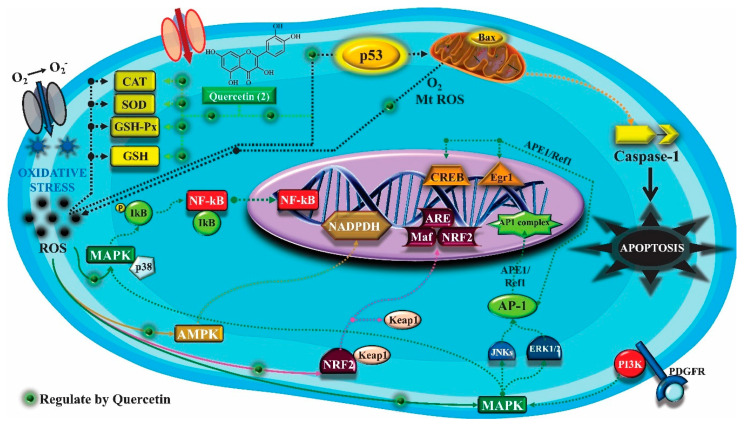
Antioxidant effect of quercetin on enzyme activity, signal transduction pathways and reactive oxygen species (ROS). Several conditions and environmental factors can increase ROS production. Besides, the mitochondrial electron transport chain is an important source of intracellular ROS generation. Flavonoid uptake occurs either via passive diffusion through cell membrane, or through membrane bound transport proteins [54]. After entering the cell, quercetin acts through the regulation of the enzyme-mediated antioxidant defense system and the non-enzymatic antioxidant defense system. Nuclear factor erythroid 2–related factor 2 (NRF2), AMP-activated protein kinase (AMPK), and mitogen-activated protein kinase (MAPK) pathways induced by ROS to promote the antioxidant defense system and maintain oxidative balance can also be regulated by phenolic compounds such as quercetin [59]. Through the neutralizing effect of ROS, quercetin can develop important anti-inflammatory effect due to inhibition of the Nuclear Factor-kappa B (NF-KB) pathway, preventing the activation of NRLP3 inflammasome (shown in Figure 1B). Through the p53 pathway, ROS induce apoptotic events. Therefore, quercetin can prevent apoptosis induced by excess ROS. In addition, it enhances the production of Apurinic/apyrimidinic Endonuclease 1/ Redox Effector Factor 1 (APE1/Ref1), activation of various signaling events and the NF-E2-related factor (NRF2)-mediated activation of genes, containing antioxidant response elements (ARE) and NF-κB [60,61,62,63,64]. AMPK, AMP-activated protein kinase; AP-1, activator protein 1; APE1, Apurinic/apyrimidinic endonuclease 1; ARE, antioxidant response element; Bax, BCL2 Associated X; CAT, catalase; CREB, cAMP-response element binding protein; EGR1, Early Growth Response 1; ERK, Extracellular signal-regulated kinase; GSH, glutathione; GSHPx, Glutathione peroxidase; IκB, κB inhibitor; JNK, c-Jun N-terminal Kinase; KEAP1, Kelch-like ECH-associated protein 1; Maf, musculoaponeurotic fibrosarcoma; MAPK, mitogen-activated protein kinase; MtROS, Mitochondrial ROS; NF-ΚB, Nuclear Factor-kappa B; Nrf2, nuclear factor erythroid 2–related factor 2; PDGFR, Platelet-derived growth factor receptors; PI3K, phosphatidylinositol-3-kinase; Ref-1, redox effector factor 1; ROS, reactive oxygen species; SOD, Superoxide dismutase.

In addition, quercetin (**2**) also has antioxidant activity, which is mainly mediated by its effects on glutathione (GSH), enzyme activity, signal transduction pathways and ROS. Increase in GSH levels in rats was observed after quercetin administration (**2**), which increases the antioxidant capacity of these animals, since GSH acts as hydrogen donor in the reaction of conversion of hydrogen peroxide into water, catalyzed by superoxide dismutase (SOD), reducing its toxicity. Quercetin (**2**) also acts by increasing the expression of endogenous antioxidant enzymes, including catalase (CAT) and glutathione peroxidase (GPx). In signal transduction pathways, quercetin (**2**) acts in the regulation of kinase protein activated by AMP (AMPK) and MAPK, stimulated by ROS, promoting antioxidant defense and maintaining the oxidative balance, since ROS lead to the activation of several pro-inflammatory and apoptotic signaling events mediated by p53, a cell cycle regulatory protein (Figure 2). Also, quercetin (**2**) inhibits the p38MAPK/inducible nitric oxide synthase (iNOS) signaling pathways, negative regulation of NF-κB levels and positive regulation of SOD activity to promote antioxidant activity [59].

*p*-Coumaric acid (**8**), identified in *T. esculenta* fruits, presents a phenyl hydroxyl group in its molecular structure, capable of promoting the neutralization of free radicals such as superoxide anion 2,2-diphenyl-1-picrylhydrazyl-hydrate (DPPH) and hydrogen peroxide (H2O2). This antioxidant property is intensified after conjugation with quinic acid (**9**), also found in *T. esculenta* fruits. In addition, *p*-coumaric acid (**8**) also has antimicrobial activity tested against three Gram-positive bacteria (*Streptococcus pneumonia, Staphylococcus aureus* and *Bacillus subtilis*) and three Gram-negative bacteria (*Escherichia coli*, *Shigelladys enteriae* and *Salmonella typhimurium*), by increasing the permeability of the bacterial membrane and binding to the phosphate anion of the DNA (deoxyribonucleic acid), altering the processes of bacterial transcription and replication [65].

In a previous study, researchers analyzed the antioxidant properties of *T. esculenta* fruits using two tests, the scavenging of DPPH radicals and the iron reduction capacity. Antioxidant activity was detected in the seed extract, in which naringenin (**7**), luteolin (**6**) and rutin (**10**) flavonoids were identified and also for pulp extracts, where phenolic compounds such as gallic acid (**5**), *p*-coumaric acid (**8**), rutin (**10**), catechin (**3**), epicatechin (**4**) were also found, as well the cyclitol quinic acid (**9**), to which antioxidant activity can be attributed [33].

Flavonoids mirecetin (**1**) and quercetin (**2**), also found in *T. esculenta* fruits, have significant antiproliferative activity, suggesting a chemopreventive and anti-tumor potential that should be investigated in the future [32]. [35] reported two other properties of *T. esculenta* phytochemicals, diuretic and antihypertensive. Studies have shown that the hydroalcoholic extract obtained from *T. esculenta* leaves and stem promotes significant increase in urinary volume, without changing urine pH and density, indicating a diuretic effect, and significant increase in renal potassium elimination, which are properties related to phenolic acids and flavonoids found in extracts.

Pinheiro et al. [66] analyzed the possible antifungal activity of lectin extracted from *T. esculenta* seeds. This activity was tested on *Microsporum canis*, a filamentous keratinophilic fungus that causes infections in skin, hair and nails in humans and animals. The results show the ability of lectin to inhibit the growth of the fungus, which may be associated with the interaction of lectin with carbohydrates on the surface of microorganism such as d-mannose and *N*-acetyl-glucosamine, since the addition of these carbohydrates caused inhibition of the antifungal effect, probably due to competition for the interaction of fungal carbohydrates with lectin. The ability of lectin to inhibit the adherence of microorganisms and exert antimicrobial effects was analyzed in another study, which tested such properties on bacteria *Streptococcus mutans* UA159, *Streptococcus sobrinus* 6715, *Streptococcus sanguinis* ATCC10556, *Streptococcus mitis* ATCC903 and *Streptococcus oralis* PB182. The results indicate that lectin was not able to inhibit the growth of bacteria at any of the applied concentrations and also did not inhibit the adherence of microorganisms, that is, it does not present antimicrobial activity and does not inhibit biofilm formation [67].

#### 3.1.5. Toxicity Studies

So far, there are no records of human poisoning by *T. esculenta*, probably because seeds and leaves are not consumed in the fresh form, only the pulp; however, there are records of intoxication in some animals, such as sheep and cattle that ingest leaves and seeds without heat treatment, which may indicate that the toxic compound is affected by high temperatures [68].

The Northeastern region of Brazil presented an outbreak of spontaneous poisoning in sheep and cattle, which showed severe signs of nervous system damage, some irreversible. Thus, an experimental reproduction of the poisoning was carried out with 5 sheep by administering 30–60 g of leaves per kilogram of body weight, and two sheep with doses of 5–10 g of seeds per kilogram of body weight, with samples from different regions of Brazil. All sheep showed clinical signs of intoxication 72 h after exposure. The main signs and symptoms were mild to moderate tympanism, drowsiness, ataxia, depressive behavior and humeral hypomotility. The chemical compound present in leaves and seeds responsible for the toxic effect is still unknown. In addition, the minimum amount that exerted toxicity was lower for seeds than for leaves, indicating greater potential for seed toxicity [69].

In the same region, cattle spontaneously intoxicated with the same clinical signs, were analyzed 72 h after exposure. In autopsy exams, partially digested seeds and leaves were found in the rumen. Laboratory and histological exams showed no significant changes, either in spontaneous or experimental poisoning. However, the presence of seeds in the rumen content associated with clinical signs suggests that there is risk of human poisoning by both seeds and leaves [70]. Similar clinical signs were observed in a dog after ingestion of *T. esculenta* seeds. Although the substance responsible for the toxicity is unknown, the induction of inflammatory response by lectin found in seeds is the focus of a study that describes the recruitment of neutrophils and mononuclear cells caused by lectin. The proposed mechanism is related to the specific properties of lectin binding to carbohydrates in the cell membrane [71,72].

In contrast, Wistar rats treated with purified aqueous extract from *T. esculenta* leaves and stem at oral doses of 5, 50, 300 and 2000 mg did not show any sign of acute toxicity, and regardless of dose, there were no abnormal signs in comparison with control animals. Water intake and body weight did not change during the experimental period and the biochemical and hematological parameters showed no abnormalities. After euthanasia, heart, lung, spleen, kidney and liver samples were collected for pathological evaluation and also showed no signs of abnormality, which may be related to the lectin concentration, which is higher in seeds than in leaves and stem; therefore, the absence of toxicity may be due to the fact that seeds were not used in this study [35].

## 4. Moraceae Family

The Moraceae family has 53 genera and about 1500 identified species, with tropical prevalence, being more than 50% of the genera present in the Neotropical region, mainly in South America. Species of the Moraceae family are found in humid forests or in their vicinities. *Artocarpus, Brosimum, Ficus* and *Morus* are among the most widely known genera, which correspond to the widely known and consumed fruit-producing plants of great nutritional and economic importance such as jackfruit, walnut, fig, and blackberry. In addition, some species of this family provide wood and leaves, used as food for silkworm [11,73,74].

Belonging to the Rosales order, this family has members that stand out for their ornamental possibilities, such as the genus *Ficus, Maclura* and *Dorstenia* and medicinal possibilities such as *Brosimum gaudichaudii* Trécul. This family was classified as the most important in terms of number of species with phytotherapic potential, emphasizing the genus *Brosimum* [75,76].

Like other families characteristic of tropical climates, Moraceae members also present adaptations to water scarcity and intense solar radiation. An example of these adaptations is the development of a thick waxy layer, called cuticle, with significant photoprotective property, which due to its reflective capacity, prevents the intense and harmful absorption of excessive solar radiation [77].

### 4.1. Genus Brosimum and Species Brosimum gaudichaudii

The genus Brosimum is composed of 13 species: *Brosimuma cutifolium* Huber, *Brosimum alicastrum* Sw., *Brosimum gaudichaudii* Trécul, *Brosimum glaucum* Taub., *Brosimum glaziovii* Taub., *Brosimum guianense* (Aubl.) Huber, *Brosimum lactescens* (S. Moore) C.C. Berg, *Brosimum longifolium* Ducke, *Brosimum melanopotamicum* C.C. Berg, *Brosimum parinarioides* Ducke,* Brosimum potabile* Ducke, *Brosimum rubescens* Taub., and *Brosimu mutile* (Kunth) Pittier, with *B. gaudichaudii* being the only representative of the genus Brosimum found in the Cerrado vegetation [78,79].

There are several studies focused on the biological properties of *Brosimum gaudichaudii* Trécul, which has great economic importance due to the production of latex, in addition to the use of roots, stem bark and leaves in popular medicine, whose pharamacological activities are attributed to the high content of coumarins, its main class of active metabolites, which may represent about 3% of the dry root weight [80,81,82].

Popularly known as mama-cadela, mamica de cadela, conduru and inharé, this species has properties of agribusiness interest, contributing to the economic development of the country. Its wood is used in civil construction and paper-making industries, and although its fruit is edible, the use of roots, stems and leaves in popular medicine prevails [73].

#### 4.1.1. Geographical Distribution and Popular Use

*B. gaudichaudii* is not endemic to Brazil, despite being widespread in the country, as it occurs in other countries, mainly in Paraguay, Bolivia, and Argentina. It is predominant in regions with typical Cerrado, Cerradão and Amazon savanna vegetation. However, it is under threat of extinction due to its occurrence in regions with constant change, such as the Brazilian cerrado, whose native vegetation is commonly subjected to burning and reduced by the expansion of the agricultural frontier, in addition to being affected by the indiscriminate extraction of latex [83,84].

Ribeiro et al. [85] conducted a survey on the popular use of several species, among them *B. gaudichaudii*, whose most used plant organs were roots, stem bark and latex, either in the fresh form or prepared by decoction, infusion, or maceration. The therapeutic indications reported in the research were: infections, venereal diseases, that is, those sexually transmitted, boils, superficial skin mycoses, cancer, anemia, cardiac arrhythmia, pneumonia, vitiligo, joint pain, inflammation, rheumatism, kidney disorders and wound healing.

For the treatment of vitiligo and other skin diseases, the extract, usually obtained by infusion or decoction of roots or stem bark, is used topically. In addition, it has also been described as a depurative, that is, it is used to eliminate toxins and improve blood circulation, and in this case, preparation is carried out by decoction or maceration of branches and leaves in dry wine. Its use against flu, colds and bronchitis occurs from the infusion of any plant part in wine or water [86].

Popularly known as bottleful, homemade preparations using dry wine as vehicle are produced by macerating the chosen vegetable organ in wine and honey for a period of at least 8 days. After this period, the liquid obtained is packed in capped bottles. Generally, extracts are orally administered and although they do not have a health record, they can be found available for purchase at street markets [87,88].

#### 4.1.2. Botanical Aspects

*B. gaudichaudii* can be found as shrub, tree or bush, reaching up to 4 m in height. Roots are terrestrial and gemiferous, that is, with the ability to sprout and generate new plants, a strategy of survival to common fires that occur in the Cerrado region, they form a root system, composed of a main root to which longitudinal roots that grow in different directions are anchored. The stem is aerial, erect and of the trunk type, with sympodial growth; leaves are glabrous on the adaxial surface and may present glandular trichomes on the abaxial epidermis, phyllotaxis is alternate, with simple and petiolate leaves, with oblong, oblong-lanceolate or elliptical shape, obtuse to acuminate apex, oblique base, entire margins of the limb, wavy or serrated, with camptodrome-like peninerval venation, that is, secondary venation do not end at the margin [83].

It blooms between the months of June and October; inflorescences are monopodial, bisexual, ear-like, globose, composed of 30 to 100 flowers, which are pedunculated, aclamidic, diclinic, monoic, that is, they present unisexual female and male flowers in the same individual, and staminate flowers are protected by bracteole. They have green to yellowish color, with gamocarpelar gynoecium and unilocular ovary [89,90].

*B. gaudichaudii* fruits are edible, drupe type, fleshy, with about 2 to 4 cm in diameter, globose, monospermic, and should be harvested between the months of September and November. Each fruit contains an ellipsoid seed, whitish in color, considered recalcitrant, as it is not viable after drying. The embryo has two fleshy cotyledons and fills the entire seed volume. Regarding shape and size, these cotyledons have starch, protein, and lipid reserves. They have short and swollen hypocotyl located below the point of insertion of cotyledons [82].

#### 4.1.3. Phytochemical Aspects

The summary of phytochemical studies carried out with different *B. gaudichaudii* plant organs and the structures of isolated substances are shown in Table 2.

*B. gaudichaudii* is the target of some phytochemical screening studies, mainly qualitative, which show significant concentrations of coumarins, especially furanocoumarin psoralen (**25**) and bergaptene (**26**). The analysis of the proximate composition of the fresh ripe fruit with bark showed for every 100 g on a wet basis: 77.63 g of moisture, 1.63 g of proteins, 0.60 g of lipids, 13.35 g of carbohydrates, 5.11 g of dietary fiber, 0.82 g of ash content and 62.21 kcal of total energy value. In addition, 46.47 mgGAE (gallic acid equivalents) of phenolic compounds and 14.92 g of vitamins C were also quantified [95,96].

Lourenço, [91] identified and quantified the content of furanocoumarin psoralen (**25**) and bergaptene (**26**) in the lyophilized methanolic and aqueous extracts of the root cortex of *B. gaudichaudii*. For each 1 g of dry weight of the plant organ used, 27.6 mg of psoralen (**25**) and 32 mg of bergaptene (**26**) were found in the methanolic extract, which correspond, respectively, to 2.8% and 3% of the sample. For the aqueous extract, 7.1 mg/g of psoralen (**25**) were quantified, that is, 0.7% and 2.6 mg/g of baptapene (**26**). This study analyzed the composition of the methanolic extract of leaves, branches, latex, and heartwood of *B. gaudichaudii* roots. In leaves, glycosylated flavonoids 5,7,3’, 4’-tetrahydroxy-6-*C*-glucopyranosylflavone (**27**) and 5,7,3’,4’-tetrahydroxy-3-*O*-ß-d-galactopyranosyl-flavonol were detected (**28**), also in the extract of leaves, branches and heartwood in roots, psoralen (**25**) and bergaptene (**26**) were also isolated, but in less amount compared to that found in the root cortex, while furanocoumarins were not detected in the latex.

Other analyses using the hydroalcoholic extract of *B. gaudichaudii* roots also identified psoralen (**25**) and bergaptene (**26**). Two extraction techniques were compared, percolation and ultrasound-assisted extraction (UEA), and both showed satisfactory results, but UEA extraction technique was more quick [92].

Spectral analysis was carried out on an extract from *B. gaudichaudii* root bark, which led to the identification of a new coumarin called gaudichaudine (**29**), the coumarins psoralen (**25**), bergaptene (**26**), luvangetin (**30**) and (+)−(2’*S*, 3’*R*)-3’-hydroxyrmesin (**31**) and the pyranocoumarin xanthyletin (**32**). Subsequently, using the same type of extract and the same plant organ, the same researchers found the following secondary metabolites: coumarins marmesin (**33**), 1’,2’-dehydromarmesin (**34**), 8-methoxymarmesin (**35**) and 1’-hydroxy-3’-*O*-β-glucopyranosylmarmesin (**36**); the chalcone 2’,4’,4-trihydroxy-3’3-diprenylchalcone (**37**); and the triterpene β-amyrin (**38**) [93,94].

The determination of total tannins in the methanolic extract from *B. gaudichaudii* stem bark carried out by [97] revealed that for each 1 g of sample, there are approximately 17.50 mg of total tannins, which have not been isolated so far. Another study on the same species qualitatively identified in the alcoholic extract of leaves and stem bark, alkaloids, anthraquinones, phenols and tannins [98].

The qualitative analysis of the ethanolic extract from *B. gaudichaudii* root bark pointed out the presence of triterpenes, steroids, coumarins, alkaloids and anthraquinones, but tested negative for saponins and tannins [99]. To investigate the presence of these and other compounds in the ethanolic extract from *B. gaudichaudii* leaves, a previous study carried out a qualitative analysis of this extract, which was positive for alkaloids, coumarins, flavonoids and cardiotonic glycosides, and negative for anthraquinones, catechins and saponins [100].

#### 4.1.4. Pharmacological Studies

**Figure 3:**
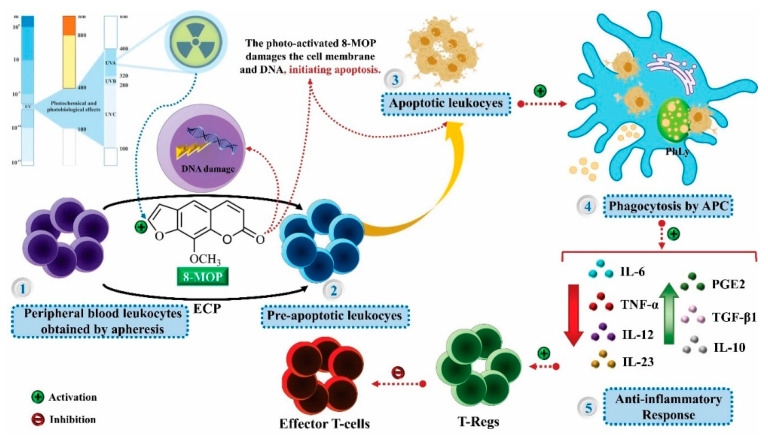
Use of furanocoumarins in the technique of extracorporeal photopheresis for the treatment of systemic or multifocal diseases: Leukocytes obtained by apheresis are exposed to 8-metoxipsoraleno (8-MOP), which is activated by UVA radiation and covalently binds to the DNA of these cells, causing damage and inducing apoptosis within 48 h. Pre-apoptotic leukocytes are reintroduced into the peripheral circulation, being recognized and phagocyted by antigen-presenting cells in phagolysosomes. This recognition induces tolerogenic anti-inflammatory response, which reduces the production of pro-inflammatory cytokines like IL-6, IL-12, IL-23, and TNF-α and increases the production of anti-inflammatory cytokines like IL-10, TGF-β1, and PGE2 [104]. 8-MOP, 8-metoxipsoraleno; IL-6, interleukin 6; IL-10, interleukin 10; IL-12, interleukin12; IL-23, interleukin 23; TGF-β1, Transforming growth factor beta 1; TNF-α, Tumor Necrosis Factor-Alpha. Another technique that uses furanocoumarins is extracorporeal photopheresis (ECP), which treats systemic or multifocal diseases such as Crohn’s disease, type 1 diabetes mellitus, multiple sclerosis, and rheumatoid arthritis. In this technique, the most used furanocoumarin is 8-methoxypsoralen (8-MOP), to which leukocytes, obtained by apheresis, are exposed. 8-MOP is activated by radiation and covalently binds to leukocyte DNA, leading to apoptosis within 48 h. These pre-apoptotic leukocytes are reintroduced into the peripheral circulation, where are recognized and phagocyted by antigen-presenting cells in phagolysosomes. This recognition induces a tolerogenic anti-inflammatory response that leads to a reduction in the production of pro-inflammatory cytokines IL-6, interleukin-12 (IL-12), interleukin-23 (IL-23) and TNF-α and induces the production of anti-inflammatory cytokines such as interleukin-10 (IL-10), transforming growth factor beta 1 (TGF-β1) and prostaglandin E2 (PGE2) (Figure 3) [102,104].

The pharmacological use of *B. gaudichaudii* is widely explored due to its repigmentating property, clinically used for the treatment of vitiligo, a skin pigmentation anomaly. This property is attributed to the presence of furanocoumarins, molecules found mainly in the *B. gaudichaudii* root cortex, which have photosensitizing action, and associated with ultraviolet (UV) radiation, is used to treat not only vitiligo, but also other skin disorders such as psoriasis, systemic lupus erythematosus and mycoses [101] (Figure 3).

The mechanism of action of furanocoumarins for the UV phototherapy technique is not yet defined. However, previous studies have proposed that there is induction of apoptosis of cells that participate in the generation of disorders, such as T cells, mast cells and keratinocytes, in addition to increased proliferation of melanocytes and reduced histamine release by mast cells and basophils [102]. Lang et al. [103] reported an increase in CD8 + T lymphocytes specific for the skin in a patient with vitiligo and that this may be related to the etiology of the disease, since these cytotoxic T lymphocytes may participate in the reduction in the number of melanocytes observed in the disease (Figure 3).

Lourenço, [105] tested the possible antibacterial activity of the ethanolic extract of *B. gaudichaudii* leaves and stem bark against *S. aureus* from clinical samples, beta-hemolytic streptococci, *P. aeruginosa*, *E. coli*, *Citrobacter* sp. and *Proteus* sp. Both extracts showed antimicrobial activity against all bacterial strains, including multi-resistant strains, with variable percentage of bacterial growth inhibition. However, the bioactive compounds responsible for this activity have not yet been identified.

The ethanolic extract from *B. gaudichaudii* stem bark was analyzed for antifungal properties on *Candida albicans* and *Candida* sp. This activity was observed for *C. albicans* at concentration of 200 mg/mL and for *C. non albicans* at concentrations of 100 mg/mL and 200 mg/mL [96]. The hydroalcoholic extract from *B. gaudichaudii* leaves was also tested for trypanocidal activity. Mice previously infected with the blood form of *Trypanosoma cruzi* received the extract at different concentrations. The infection was assessed by counting parasites present in 10 μL of blood between the 5th and the 11th day after infection, showing reduction in the number of trypomastigotes at concentration of 100 mg/kg. Although the chemical compound responsible for this activity has not been identified, it is possible to associate it with furanocoumarins, which are the main components of the extract that may have acted through the production of oxide and superoxide radicals [106].

β-Amyrin (**38**), a triterpene found in *B. gaudichaudii* root bark promotes sleep modulation through the activation of the GABAergic system. For the analysis, a pentabarbital-induced sleep model was used in mice, and it was observed that, after the administration of 1, 3 or 10 mg/kg of β-amyrin (**38**), the time for the beginning of sleep was reduced and the sleep duration increased significantly, which effects were inhibited after administration of a type A gamma-aminobutyric acid antagonist receptor (GABA_A_), demonstrating that this property is associated with the GABAergic system. In addition, the levels of gamma-aminobutyric acid (GABA) in the brain were analyzed, which increased after the administration of β-amyrin (**38**), which could be related to the blocking of GABA transaminase, inhibiting GABA degradation and, consequently, increasing its available concentration [107].

A study was carried out on the role of β-amyrin (**38**) in attenuating the neurodegeneration of Parkinson’s disease using *Caenorhabditis elegans* strain. The analysis describes a protective effect of β-amyrin (**38**) against neurotoxicity induced by 6-hydroxydopamine (6-OHDA), which was related to the possible antioxidant role of β-amyrin (**38**). This study also investigated its anti-apoptotic activity on the expression of pro-apoptotic genes in *C. elegans*, which were not significantly altered after treatment with β-amyrin (**38**). The aggregation of α-synuclein protein is one of the mechanisms associated with Parkinson’s disease. The effects of β-amyrin (**38**) on this mechanism were compared to the effect of the medicine clinically used to treat the disease, L-dopa and both substances significantly reduced the α-synuclein aggregation [108].

Sunil et al. [109] analyzed the antioxidant activity of β-amyrin (**38**) in Wistar rats with oxidative stress induced by carbon tetrachloride (CCl_4_). The effect was positive in the elimination of DPPH radicals, hydroxyl, nitric oxide (NO), superoxide radicals and had strong reducing and suppressive power of lipid peroxidation. The increase in free radical levels also leads to elevated levels of hepatic enzymes serum glutamic oxalacetic transaminase (SGOT), serum glutamic pyruvic transaminase (SGPT) and LDH, which after administration of β-amyrin (**38**), had their levels reduced. In addition, the levels of the antioxidant enzymes SOD, CAT, GSH and GPx, were high.

A study analyzed the β-amyrin (**38**) activity on inflammation induced by lipopolysaccharide (LPS) and IFN-γ in the microglial cells of rats. β-amyrin (**38**) reduced the expression of pro-inflammatory factors such as TNF-α, IL-1β, IL-6, PGE-2 and cyclooxygenase-2 (COX-2) and increased the expression of arginase-1. The reduction of factors was attributed to the activation of the cannabinoid receptor, since antagonists of these receptors inhibit the anti-inflammatory effects of β-amyrin (**38**). Through another not yet identified mechanism, β-amyrin (**38**) reduces the activity of COX-2 and, consequently, the production of PGE-2, commonly inhibited by the action of non-steroidal anti-inflammatory drugs. Enzymes arginase-1 and NO synthase compete for the same substrate, L-arginine, with the overexpression of arginase-1, observed by the increase in its product, urea, NO production is compromised, since the availability of substrate for NO synthase is reduced [110].

Marmesin (**33**), a coumarin found in *B. gaudichaudii* root bark, is the target of several studies that investigate its medicinal properties, especially its anti-tumor properties. The in vitro and in vivo activity of marmesin (**33**) was evaluated on cells with human leukemia and healthy human monocytes. The results indicate that marmesin (**33**) exerts dose-dependent anti-tumor activity and that the cytotoxic effect on healthy monocytes was lower, which is essential for the safety of a probable treatment, since it predicts that the compound has action selectivity, reducing the possibility of adverse effects. Marmesin (**33**) increased the expression of pro-apoptotic protein Bax and reduced the expression of anti-apoptotic protein Bcl-2, increasing the Bax/Bcl-2 ratio, which promotes activation of caspase 3, leading to apoptosis, with mechanism of action similar to other chemotherapeutic drugs. Marmesin (**33**) also causes an increase in intracellular ROS and reduces the mitochondrial membrane potential (MMP), which was related to the reduced migration of cells with leukemia, important in inhibiting metastases [111].

Another analysis of the same property associated with marmesin (**33**), but on lung cancer and tumor angiogenesis, reports anti-tumor activities of marmesin (**33**) mediated by the inactivation of mitogenic signaling pathways and negative regulation of proteins related to cell signaling, including vascular endothelial growth factor-2 receptor (VEGFR-2), integrin β1, integrin-linked kinase (ILK) and matrix metalloproteinases-2 (MMP-2), nullifying mitogen-stimulated proliferation and invasion in cells expressing p53 or not [112].

#### 4.1.5. Toxicity Studies

Furanocoumarins, the major constituents of *B. gaudichaudii* roots, are phototoxic substances that the plant uses as a protection mechanism against phytopathogenic microorganisms and herbivores. The mechanism of action is intercalation in the double helix of the DNA structure and in molecular complexation, and when activated by light, they react with pyrimidine bases, mainly with thymine, which can promote toxic, mutagenic and carcinogenic effects [113].

The genotoxic activity of *B. gaudichaudii* was evaluated using aqueous and methanolic extracts on *Salmonella typhimurium* strains, which showed an increase in chromosomal aberrations in cultures submitted to the methanolic extract in the G1/S and S phases of the cell cycle. For the aqueous extract, results were not significant, which is due to the lower amount of furanocoumarins extracted by the aqueous extract compared to the methanolic extract [114].

Lourenço, [91] performed toxicity tests for the aqueous and methanolic extract from *B. gaudichaudii* root cortex and obtained through the MTT technique (3-[4,5-dimethylthiazol-2-yl]-2,5-diphenyl tetrazolium bromide), cytotoxicity indexes (IC_50_) of 4.65 mg/mL and 1.31 mg/mL, for each extract, respectively. The mutagenicity assay was also performed with *Salmonella typhimurium*, which generated the mutagenicity ratio (MR) through the observation of revertants, that is, the number of individuals whose natural phenotype induced by mutation, was restored. The ratio is calculated using the average number of revertants in the test plate, spontaneous or induced, divided by the average number of revertants per negative control plate, that is, spontaneous, with the sample being considered positive with ratio greater than or equal to 2. MR was higher for the aqueous and methanolic extracts in the TA102 strain in the presence of microsomal fraction S9, which reveals whether the substance or sample is mutagenic in its original form or whether it needs to be metabolized or activated to become mutagenic, in this case, it has been metabolized.

A study determined the acute toxicity of the extract from *B. gaudichaudii* root bark orally and intraperitoneally administered in mice. Oral administration led some animals to death, and the median lethal oral dose (LD_50_) was 3517.54 mg/kg and intraperitoneally, LD_50_ was 2871.76 mg/kg. Up to 2000 mg/kg, 40% of animals had diarrhea, and increasing the dose, some of them presented, in addition to diarrhea, dry eyes, eyelid occlusion, ocular hemorrhage, epistaxis, weight loss, tachypnea and death at the lethal dose. Dead animals were analyzed and showed dilation and hemorrhage, in addition to increase in the amount of hemosiderin in the spleen, indicating previous hemorrhage and destruction of erythrocytes [80].

Using dry *B. gaudichaudii* extract, the acute and subacute toxicities of this species were analyzed, with an estimated lethal dose (DLA) of 3800 mg/kg. In the acute study, 14 days after administration, leukopenia and hemosiderin accumulation were observed in the spleen, and in the subacute, after 30 days, changes in the levels of aspartate aminotransferase (AST), alanine aminotransferase (ALT), creatinine and total proteins were observed, indicating hepatotoxicity and nephrotoxicity for the dose of 100 mg/kg, in addition to leukopenia and renal hemorrhage [115].

**Figure 4:**
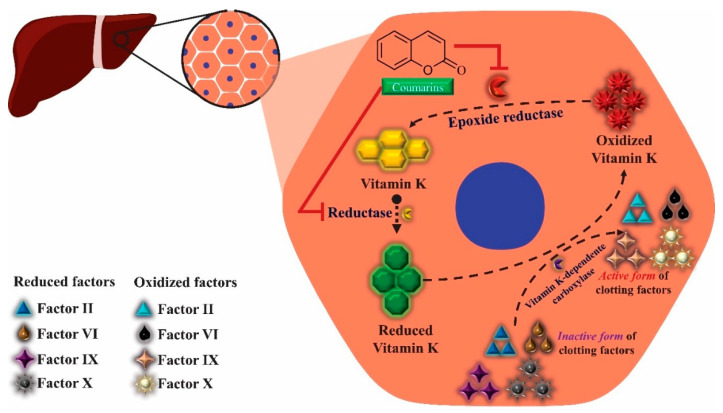
Proposed mechanism for inducing hemorrhage by coumarins: Coumarins act as competitive epoxide reductase inhibitors. This enzyme reduces oxidized vitamin K during its participation as co-factor in the synthesis of coagulation factors II, VII, IX, and X. With epoxide reductase inhibition, the reduction that occurs to regenerate vitamin K is blocked, depleting its levels and, consequently, inhibiting the synthesis of coagulation factors, causing hemorrhage.

These clinical signs of hemorrhage may be related to the anticoagulant property of some coumarins found in large amounts in this species. To exert such activity, coumarins act as competitive inhibitors of epoxide reductase, an enzyme that reduces vitamin K, oxidized by participating as co-factor in the synthesis of coagulation factors II, VII, IX and X. With the inhibition of epoxide reductase, this regeneration does not occur, depleting the levels of active vitamin K and consequently inhibiting the synthesis of coagulation factors (Figure 4) [116].

## 5. Rubiaceae Family

The Rubiaceae family is the fourth largest family of angiosperms, composed of about 650 genera and more than 13,000 species. Belonging to the order Gentianales, it is a cosmopolitan and pantropical family distributed as herbs, shrubs, lianas, small and large trees. It has great biodiversity, being of paramount importance in floristic formation, in addition to the conservation of tropical vegetation [117].

This family includes important species for the economy of several countries, being widely used as food, such as *Coffea arabica*; in popular medicine such as *Cinchona* sp.; in civil construction such as species of the genera *Sarcomphalus, Mitragyna*, *Morindae Pausinystalia*; and in ornamentation such as *Gardenia* spp., *Ixora* spp. and *Mussaenda* spp. [118].

Most fruits produced by species of this family have high levels of iridoids, which act as plant defenders against the attack of herbivores. These substances make up pigments found in extracts of species of this family and which are used as cosmetics due to their pigmenting property for the dyeing of keratin fibers [119].

### 5.1. Genus Genipa L. and Species Genipa americana L.

*Genipa* L. is a neotropical genus with species distributed in several countries, mainly on the American continent. *Genipa americana* L. is a fruit species characterized by high survival rate against drastic environmental changes, such as floods, which can cause reduction in the growth of shoots and roots, reduction in biomass and changes in their partitioning, in addition to promoting plant senescence, which promotes a mechanism of tolerance in some species frequently exposed to these conditions [120,121].

This species has been used for the regeneration of reserve areas and environmental preservation. Due to its adaptive characteristics, it can be domesticated and used in urban afforestation and agriculture, since its wood and fruits have considerable commercial value. Although little consumed in the fresh form, fruits are used for the production of jams, candies, ice cream, soft drinks, wines and liqueurs [122].

Its seeds are of easy propagation, which can be sown soon after being removed from fruits and support up to 60 days of storage, which should be performed after drying or under freezing. In addition, *G. americana* can be grown not only from seeds, but also by budding or grafting of plant parts, which produces fruit about 6 years after planting [123,124].

#### 5.1.1. Geographical Distribution and Popular Use

*Genipa americana* is widely distributed from South America to Central America. It has tropism for coastal regions and river banks, being found in the Cerrado vegetation and popularly known as jenipapo, janapabeiro, janipaba, janipapo, genipapeiro, jenipapinho, jenipá, jenipapeirol, genipapo, genipapeiro [125].

All plant organs of this species are used in folk medicine. Leaves, stem bark, fruits and roots are indicated for cough, anemia, bruises, dislocations, as depurative, cathartic, purgative, febrifuge, aphrodisiac, diuretic, for spleen and liver diseases, jaundice, and injuries. The pulp of the fresh fruit is indicated for diabetes and liver diseases, and the fruit juice is indicated for anemia [126,127,128].

For dermatitis, pulp and heated seeds are applied over the affected area. The use of tea from fruits and leaves produced by decoction for 1 h is described for the treatment of anemia, and tea from roots and stem bark is used as aphrodisiac. Tea from toasted stem bark obtained by decoction is applied in the form of poultice on bruises, fractures and twists [129,130,131].

Other forms of fruit use are reported for the treatment of anemia such as juice, bottleful, and dye. The juice is obtained after crushing the fresh vegetable organ and in its homemade preparation a pestle or blender is used, the former being more used for less juicy parts. The dye is a concentrated extract of medicinal plants and consists of an alcoholic or hydroalcoholic preparation in which the macerated plant is immersed in the extraction liquid for 8 to 10 days, and after that period the mixture is filtered, packed in a capped flask and used in the form of drops dissolved in water or used in ointments [132,133,134].

#### 5.1.2. Botanical Aspects

*G. americana* is a tree with terrestrial, adventitious roots, aerial and erect stem of trunk and circular type and sympodial growth. Leaves are simple, petiolate, with opposite phyllotaxis with obovate, elliptical-obovate or oblanceolate shape, acuminated limb apex, cuneiform base and smooth margin, with gland trichomes on the abaxial epidermis and pinnate venation [120,135].

Flowering occurs between October and December; inflorescences are dichasium with about 16 flowers, which are hermaphroditic, white to yellowish in color, aromatic, pedunculated, pentamera, with bilocular ovary. Fruits are subglobous, polyspermic, indehiscent berries, with brown color when ripe and thin pericarp. Maturation usually occurs between the months of May and August [136,137].

Seeds have ovoid shape, flat, with rigid seed coat and dark brown in color and orthodox, that is, unlike recalcitrant seeds, the degree of humidity does not imply loss of viability and are resistant to low temperatures, so drying of seeds up to 10% does not compromise their germination [137,138].

#### 5.1.3. Phytochemical Aspects

Table 3 presents a summary of phytochemical studies of *G. americana* extracts, as well as the structures of their phytochemical constituents.

The chemical characterization of *G. americana* fruits and leaves presents iridoids as major constituents, belonging to the class of terpenes. Genipin (**55**) is the main iridoid found in *G. americana* fruits and has considerable economic potential due to its pigmentation property. The extraction of genipin (**55**) can occur by three different methods, enzymatic hydrolysis, extraction with solvents and ultrasound. A study performed the extraction of genipin (**55**) from *G. americana* fruits using the enzymatic method and quantified 7.85 mg/g of this phytochemical in the sample [119,145].

Pacheco et al. [146] carried out a study on the nutritional composition and energy value of the pulp of *G. americana* fruits. After triplicate analysis, 70% of moisture, 0.5% of proteins, 0.0% of lipids, 1.1% of ash, 22.1% of carbohydrates, 6.3% of dietary fiber, 0.0 mg/100g of beta-carotene, 22.5 mg/100g of vitamin C, 176 mg GAE/100g of phenolic compounds and 90.7 kcal/100g of total energy value were found.

In the methanolic extract of *G. americana* fruits, the following iridoid glycosides were identified, isolated and structurally elucidated: geniposidic acid (**40**), geniposide (**48**), gardenoside (**54**), genipin-gentiobioside (**49**) and 4 new iridoids not previously identified: genameside A (**50**), genameside B (**51**), genameside C (**52**) and genameside D (**53**) [140]. Also in *G. americana* fruits, iridoids geniposidic acid (**40**), gardenoside (**54**), genipin-1-β-gentiobioside (**49**), geniposide (**48**), 6″-*O*-*p*-coumaroyl-1-β-gentiobioside geniposidic acid (**59**), 6″-*O*-*p*-coumaroylgenipin gentiobioside (**60**), genipin (**55**), 6′-*O*-*p*-coumaroyl-geniposidic acid (**61**), 6′-*O*-feruloylgeniposidic acid (**62**) were found in the methanolic extract from the endocarp and mesocarp. In addition, possible antioxidant and antiproliferative properties have been attributed to the extract and, mainly, to genipin (**55**) [143].

After extraction by pressurized ethanol and analysis of the genipin (**55**) and geniposide (**48**) content in the whole fruit and its parts separately at 50 °C and pressure of 2 bars, 20.7 mg/g of genipin (**55**) and 59 mg/g of geniposide (**48**) were found in the mesocarp; 1.16 mg/g of genipin (**55**) and 0.06 mg/g of geniposide (**48**) were found in seeds; 7.5 mg/g of genipin (**55**) and 39.9 mg/g of geniposide (**48**) were found in fruit bark; 38.9 mg/g of genipin (**55**) and 0.01 mg/g of geniposide (**48**) were found in the endocarp; 22.9 mg/g of genipin (**55**) and 0.1 mg/g of geniposide (**48**) were found in the endocarp extract and seeds; and 37.2 mg/g of genipin (**55**) and 0.57 mg/g of geniposide (**48**) were found in the whole fruit [144].

For fruits, the presence of other classes of secondary metabolites was investigated in the hydroalcoholic extract. Mayer reagent was used to detect alkaloids; for tannins, reaction with ferric chloride; for anthraquinones, reaction with ammonia; for flavonoids, Shinoda’s reaction; for steroids and triterpenes, the Libermann-Burchard reagent was used; the saponins test was carried out through agitation, observing the presence or absence of foam; and the coumarin test by fluorescence under UV light. The results for this qualitative analysis point to the presence of alkaloids, tannins, flavonoids, triterpenes, saponins and coumarins in the fruit pulp extract and absence of steroids and anthraquinones [147].

Silva et al. [139] identified 13 compounds in a hydroalcoholic extract of *G. americana* leaves. The following are among the isolated substances: (A) coniferin; (B) the iridoids asystasioside D (**39**), geniposidic acid (**40**), tarenoside (**41**) and teneoside A (**42**); (C) loganic, chlorogenic and 1,3-di-*O*-caffeoylquinic acids; and (D) flavonoids, first identified in this genus, kaempferol-3-*O*-hexoside-deoxyhexoside-7-*O*-deoxyhexoside (**43**), isorhamnetin-3-*O*-hexoside-deoxyhexoside-7-*O*-deoxy-hexoside (**44**), quercetin-3-*O*-hexoside-deoxyhexoside (**45**), kaempferol-3-*O*-hexoside-deoxyhexoside (**46**) and isorhamnetin-3-*O*-hexoside-deoxyhexoside (**47**). Other iridoids were also found in the hydroalcoholic extract of *G. americana* leaves such as genipin derivative (**55**), 1-hydroxy-7-(hydroxymethyl)-1*H*,4a*H*,5*H*, 7a*H*-cyclopenta[c]pyran-4-carbaldehyde (**56**) and 7-(hydroxymethyl)-1-methoxy-1*H*,4a*H*,5*H*,7a*H*-cyclopenta[c]pyran-4-carbaldehyde (**57**) [142].

There are few studies on phytochemical screening and characterization of compounds for *G. americana* stem, roots, and seeds. A qualitative study that analyzed the ethanolic extract of leaves and stem bark determined the presence of flavonoids, xanthones, saponins and triterpenes in the stem bark and saponins and triterpenes in leaves [148]. [149] isolated and characterized lectin present in *G. americana* stem bark, which was named GaBL and tested for hemagglutinating properties.

Neri-numa et al. [143] evaluated the antioxidant and antiproliferative activity of the methanolic extract from *G. americana* ripe and green fruits. The ability to eliminate DPPH radicals has been reported to be like that of ascorbic acid, and the extract from green fruits has higher concentration of iridoids and greater efficiency to eliminate radicals. The *in vitro* antiproliferative activity was also observed with efficiency in all tested cell lines, with greater activity for the extract from green fruits, which has high concentration of iridoid genipin (**55**), to which this property was attributed. In addition, the anticholinesterase activity of the ethanol extract from *G. americana* fruit bark, pulp and seeds was also observed and may be associated with the presence of chlorogenic acid, an acetylcholinesterase (AChE) inhibitor [139,150].

Genipin (**55**), a triterpene found in *G. americana* fruits was tested *in vitro* and *in vivo* for its anti-inflammatory property and its role on memory deficiencies induced by LPS. Microglia stimulation by LPS of gram-negative bacteria induces the production of inflammatory mediators, whose overproduction can cause neuronal damage. Genipin (**55**) inhibited the production of these mediators in the BV2 microglial cell line through the dose-dependent suppression of LPSe-induced NF-κB activation by activating the expression of erythroid nuclear factor 2 (Nrf2) and heme oxygenase-1 (HO-1). Active NF-κB induces the production of inflammatory mediators such as PGE2, TNF-α and IL-1β, while Nrf2 encodes antioxidant enzymes such as HO-1, which promote the elimination of ROS and, consequently, the inhibition of the NF-κB expression [151].

The anti-inflammatory role of genipin (**55**) is also associated to another mechanism, the inhibition of the activation of NLRP3 and NLRC4 inflammasomes by suppressing macrophage autophagy. Agonists of NLRP3 and NLRC4 inflammasomes promote the activation of autophagy in macrophages, which enhances the secretion of IL-1β and ASC oligomerization, while suppression of autophagy promoted by genipin (**55**) inhibits this effect [152].

The protective role of genipin (**55**) on LPS-induced acute lung injury has been investigated. Genipin (**55**) positively regulated the signaling of the phosphoinositide 3-kinase/phosphorylated protein kinase B (PI3K/p-AKT) pathway by increasing the levels of p-AKT. PI3K generates phosphatidylinositol-3,4,5-triphosphate (PIP3), which acts as a second messenger and facilitates the translocation of protein kinase B (AKT) to the plasma membrane, where it is activated by phosphorylation and can later be transported to the nucleus. AKT promotes the phosphorylation of some molecules, among them AMPc-responsive binding protein (CREB), whose activation is associated with increased Bcl-2 activity, which inhibits pro-apoptotic caspase-9, promoting a protective effect of cell survival [153,154].

In contrast, the action of genipin (**55**) on the PI3K/p-AKT pathway was also related to its inhibitory activity on the growth of human bladder cancer cells [155] and squamous cell carcinoma [156]. It was observed that genipin (**55**) induced the cell cycle to stop in G0/G1 phases, and promoted the apoptosis of cancer cells, with increase in the expression of pro-apoptotic protein Bax. Such cell growth suppressive effects have been associated with inactivation of the PI3K/p-AKT pathway, shown by the reduction of phosphorylated PI3K and AKT levels [155].

Zhao et al. [157] analyzed the protective role of genipin (**55**) against ischemia-reperfusion lesion associated with energy deficiency and oxidative stress, which are regulated by mitochondrial uncoupling protein 2 (UCP2) and NAD-dependent deacetylase sirtuin-3 (SIRT3), respectively. In this lesion, damage increases due to the increase in the ischemia duration, as well as the degree of energy deficiency and oxidative stress, with increase in UCP2 expression and SIRT3 activity. Genipin (**55**) acts as a specific inhibitor of UCP2. Therefore, in mice submitted to treatment with genipin (**55**), reduction in UCP2 expression and SIRT3 activity was observed, as well as a lower NAD +/NADH ratio and increased levels of adenosine triphosphate (ATP), reducing oxidative stress and energy deficiency and, consequently, mitigating damage.

The effects of genipin (**55**) on energy metabolism are also related to its anti-tumor property, capable of inhibiting the proliferation of several cancer cells in breast [158], colon [159], hepatocellular [160] cholangiocarcinoma [161] and gioblastoma [162]. UCP2 overexpression is observed in tumor cells, which gives genipin (**55**), a UCP2 inhibitor, a potential anti-tumor activity mechanism. UCP2 promotes the decoupling of the electron transport chain to oxidative phosphorylation, reducing energy availability and the production of O_2_^−^, a ROS.

**Figure 5:**
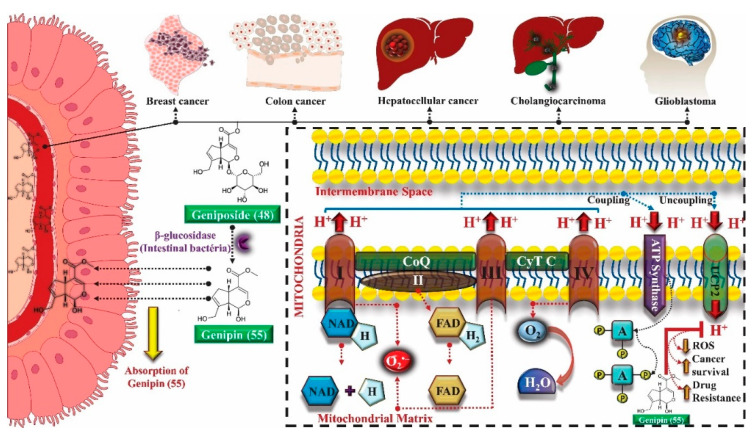
Effects of genipin on energy metabolism: anti-tumor property: Transport mechanisms of geniposide and genipin, which are abundantly present in extracts from plants such as *Genipa americana*, involve converting geniposide into genipin in the intestinal lumen through bacterial enzymes β-glucosidases. Uncoupling protein 2 (UCP2) is a genipin target in the treatment of cancer. In mitochondria, the respiratory chain, formed by complexes I to IV, transfers electrons from NADH through oxidation-reduction reactions. Complexes I, II, and III contribute to the production of H+ ion gradient. The electrochemical gradient generated is coupled to the ADP phosphorylation process via ATP synthase. Oxygen is the final electron acceptor and is reduced to water by the electron transfer of complex IV. However, its early reduction into complexes I and III leads to the formation of O_2_^•–^. UCP2 is a protein widely expressed in tumor cells. Its function is to reduce ROS production and increase the survival of tumor cells by uncoupling the electrochemical gradient generated by the respiratory chain. For this purpose, UCP2 increases H^+^ output from the intermembrane space to the mitochondrial matrix and reduces the mitochondrial membrane potential. This mechanism, present in tumor cells as a survival factor by reducing ROS generation, is the genipin target [165]. A, adenosine; CoQ, coenzyme Q; Cyt C, cytochrome C; FAD, flavin adenine dinucleotide; NAD, Nicotinamide adenine dinucleotide; ROS, reactive oxygen species; UCP2, uncoupling protein 2.

Cancer cells are under oxidative stress and to protect themselves, they increase the UCP2 expression to reduce the formation of ROS. UCP2 inhibition by genipin (**55**) promotes an increase in ROS, triggers the nuclear translocation of glycolytic enzyme glyceraldehyde 3-phosphate dehydrogenase (GAPDH), formation of autophagosomes and expression of LC3-II autophagy marker, leading to cell death or growth inhibition, invasion and migration of tumor cells. In addition, genipin (**55**) enhances autophagic cell death induced by gemcitabine, a clinically used chemotherapeutic agent (Figure 5) [163,164].

**Figure 6:**
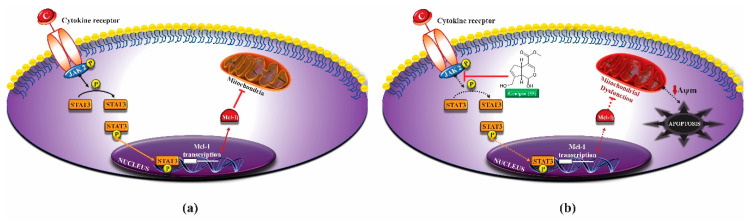
Apoptosis mediated by genipin through interference with myeloid cell leukemia-1 (Mcl-1) synthesis in gastric cancer cell lines: (**a**) Cytokine receptors without intrinsic protein kinase domain amplify extracellular signals through signal transduction via Janus Kinase (JAK) family (JAK1 to JAK3 and tyrosine kinase 2). After receptor activation, JAK2 phosphorylates the tyrosine residue of transcription factor Signal Transducer and Activator of Transcription 3 (STAT3), which enables its binding to the promoter of target genes related to survival and apoptosis. Subsequently, Mcl-1 is synthesized; (**b**) Genipin absorption by tumor cells induces mitochondrial dysfunction due to decreased Mcl-1 expression through the JAK2/STAT3 pathway. Δψm, mitochondrial membrane potential; JAK2, Janus Kinase 2; Mcl-1, myeloid cell leukemia-1; STA3, Signal transducer and activator of transcription 3 [166].

Another anti-tumor mechanism associated with genipin (**55**) occurs through negative regulation of the signal transducer and transcription activator (Stat)/cell differentiation protein of induced myeloid cell leukemia 1 (Mcl-1). Mcl-1 is a member of the Bcl-2 family and has anti-apoptotic activity, being associated with cell survival and is overexpressed in gastric cancer cells. The apoptotic mechanism of cancer cells promoted by genipin (**55**) is related to the negative regulation of Mcl-1, which can occur through the activation of the SHP-1 phosphatase and the suppressor of cytokine signaling 3 (SOCS3). In addition, this phytoconstituent inhibits the activity of JAK2 enzymes of the Janus kinase family (JAK), responsible for the activation of the Stat3 transcription factor, which regulates the expression of genes related to cell survival. Thus, inactivating JAK2 enzymes, there is no activation of Stat3 and expression of the MCL1 gene, which encodes the anti-apoptotic protein Mcl-1 (Figure 6a,b) [166].

Inhibition of Sonic Hedgehog, one of three proteins in the signal family called hedgehog found in mammals by genipin (**55**), was also associated with its anti-tumor property. Genipin (**55**) binds to the protein of the Hedgehog Smoothened (SMO) signaling pathway through the drug-affinity-responsive target stability (DARTS), increasing the expression of p53 and NOXA, a protein of the Bcl-2 family that contributes to apoptosis promoted by p53. This mechanism occurs by inhibiting the expression of the GLI1 gene, a transcriptional activator of the Hedgehog pathway that reduces p53 expression. Thus, the binding of genipin (**55**) to SMO promoter induces a reduction in GLI1 activity and an increase in p53 expression [167].

Genipin (**55**) was used in cattle to increase corneal stiffness and induce corneal collagen cross-linking (CXL), which reduces the progression of ectasia, that is, corneal distention. Through a mechanism still unknown, genipin (**55**) induces CXL by up to 7%, a result superior to that induced by treatment with riboflavin and UV light applied in the control group, which presented only 5.6% cross-linking. To achieve 7% cross-linking, 140 μL of 0.5% genipin (**55**) was administered every 1 h for 2 h [168]. The role of genipin (**55**) investigated in cattle suggests further studies in humans. Furthermore, this cross-linking property of genipin (**55**) has also been used in the biotechnological production of hydrogels, gelatin biofilms and transdermal patches for the controlled release of drugs [169].

Also, for ophthalmic treatment, genipin (**55**) was used in posterior scleral contraction/reinforcement surgery (PSCR), which delays axial stretching of the eyeball, common in human myopia. However, despite the significant effect of the procedure, it was not sustained in the long term, which was related to the loss of sclera resistance to traction. The sclera is a layer of fibrous, opaque and dense tissue that lines the eye, on which the cross-linking capacity of genipin was tested (**55**), which doubled the sclera resistance and increased by 30 % resistance to enzymatic degradation, which could promote sclera weakening. The study points out the efficacy and safety of PSCR with sclera cross-linked with genipin (**55**) to restrict axial elongation of the eyeball [168].

Genipin (**55**) was tested for possible antiviral activity on Kaposi’s sarcoma herpes virus (KSHV). Genipin (**55**) played a double and dose-dependent role. At lower concentration and administered for 48 h, the phytochemical significantly reduced the production of the nuclear antigen associated with KSHV latency (LANA) and increased the number of copies of the virus intracellular genome, favoring lytic replication of KSHV. Treatment with higher genipin (**55**) doses induced the activation of caspases 3 and 7 by reducing the expression of Bcl-2, promoting apoptosis, which is impaired, since the virus produces viral Bcl-2, approximately 60% identical to cellular Bcl-2, which makes the infected cell more resistant. New studies have been proposed to investigate the role of genipin (**55**) in modulating the KSHV life cycle and possibly prevent disorders associated with the virus [170].

Nonato et al. [171] evaluated the GABA-mediated anticonvulsant effect of the methanolic extract from *G. americana* leaves rich in polysaccharides. A heteropolysaccharide (PRE) with inhibitory and anticonvulsant effect on the central nervous system (CNS) was identified, which was reversed after the administration of the GABAergic flumazenil antagonist, indicating the participation of this receptor in the effect performed by PRE, which also reduced oxidative stress in the pre-frontal cortex, hippocampus and striated nucleus of animals that had induced seizures, observed by the increase of GSH levels and reduction of lipid peroxidation levels.

In *G. americana* leaves, a glycoconjugate rich in arabinogalactane and uronic acid was found, with anticoagulant, antiplatelet, and antithrombotic properties. Anticoagulant activity was observed in fraction containing uronic acid and occurs through the intrinsic and/or the common pathway of the coagulation cascade by a still unknown mechanism. Glycoconjugate inhibits platelet aggregation induced by adenosine diphosphate (ADP), but not collagen-induced aggregation. Antithrombotic action was observed in a model of rats with venous thrombosis and, similar to the antiplatelet activity, it was found in fraction rich in arabinogalactane [172].

The hydroalcoholic extract from *G. americana* stem bark was evaluated for its possible antimicrobial properties. Minimum Inhibitory Concentration (MIC) tests were carried out with *E. coli*, *S. aureus* and *P. aeruginosa* and MIC was ≥ 1024 μg/mL in all strains. Efficiency was not considered satisfactory, but the association of the extract with aminoglycoside drugs amikacin and gentamicin and the lincosamide clindamycin, increased the antimicrobial potential of these drugs. This property was attributed to tannins present in the extract with antimicrobial activity. The result of this association induced greater susceptibility of *P. aeruginosa* and *E. coli* to death by gentamicin and *S. aureus* by Amikacin in all strains submitted to this treatment [173].

The polysaccharide extract from *G. americana* leaves was tested against *Trypanosoma cruzi* epimastigotes, trypomastigotes and amastigotes. The results showed antiparasitic effect against the three forms of the protozoan with low toxicity to mammalian cells. In addition, it demonstrated potent activity even on amastigote forms, which are intracellular, suggesting that the compound responsible for this activity has access to the intracellular medium. After extract administration, ROS generation was observed, which causes damage to trypanothione reductase, an enzyme important for the oxidative balance of the protozoan. Morphological changes indicate cell death due to necrosis with rounding and shortening of the parasite, cytoplasmic leakage and membrane degradation [174].

#### 5.1.4. Toxicity Studies

Studies focusing on the possible toxicological effects of *G. americana* extract and its chemical constituents are still scarce. A study carried out on the acute toxicological analysis of the hydroalcoholic extract from *G. americana* fruits reported that animals receiving the extract did not present any apparent behavioral or clinical changes; the microscopic study of organs also did not present any changes, there were no deaths by the end of the study, although it was estimated that the LD_50_ would be greater than 2000 mg/kg. In the analysis of the subchronic toxicity, macroscopic changes in kidneys were described, such as increased size, which may be related to renal hyperplasia or thrombosis at dose of 100 mg/kg [175].

The toxicological evaluation of the aqueous extract from *G. americana* leaves induced mortality in *Danio rerio* fish species at concentrations above 100 mg/L. No genetic mutations were observed; however, some nuclear abnormalities such as blebbed, lobed and notched nucleus and binucleated cells were rather observed [176].

## 6. Bromeliaceae Family

The Bromeliaceae family is composed of approximately 56 genera and 3086 species. Due to the great economic potential, extraction from natural environments and, mainly, due to the ornamental value among landscapers and gardeners, some species are threatened with extinction. Species are predominantly neotropical and can be found on the American and African continents. They present great ecological diversity, with terrestrial and epiphytic species, which are arboreal, shrub or cactaceae [175,177].

One species that stands out for its economic potential is *Ananas comosus* (L) Merr, known as pineapple which, in addition to its potential for fresh consumption, is also used as a raw material to produce numerous by-products. Many plants in this family are utilized in the automotive and textile industry, such as *Ananas lucidus* Miller and *Neoglaziovia variegata* Mez due to their important properties for the production of fibers [178].

In addition, some members of the Bromeliaceae family stand out for producing large amounts of proteins and enzymes that cleave peptide bonds between amino acids in proteins. The effect of these proteins on plant physiology is not yet known, but one hypothesis is that these enzymes play the role of protecting plants against pathogens and herbivores [179].

### 6.1. Genus Bromelia and Species Bromelia antiacantha

The genus *Bromelia* comprises about 46 species distributed throughout Americas and used in folk medicine against parasitic diseases, edema, respiratory and kidney problems, intestinal disorders, and diabetes. *B. antiacantha* Bertol. is popularly known as caraguatá, gravatá, carauatá or croatá and has properties that contribute on a large scale for the economic development of the region where it occurs or is cultivated [180,181,182].

The species adapts to different climatic conditions, being found in humid and flooded soils and even in the post-beach forest, which indicates tolerance to high salinity and soaked soils. In addition, it can also be found in xerophytic environments, in which there is scarcity of water and nutrients such as Cerrado soils submitted to drought periods, in these cases, the plant adapts with the presence of a structure for storing water and nutrients, the rhizome [183].

This species has great diversity of applications, and fruits are not only used as food in the production of jellies and ice cream, but the plant is also used for ornamental purposes. Proteolytic enzymes were detected in the crude extract from *B. antiacantha* fruits and the new protease Antiacanthain A, a molecule with interesting characteristics for biotechnological use, was recently isolated [179,184]. These enzymes are used in the chemical, pharmaceutical, food and textile industries and in the production of detergent for cleaning clothes due to their stain-removing property [185].

#### 6.1.1. Geographical Distribution and Popular Use

Occurrences of *B. antiacantha* are recorded in several American countries, among them, Venezuela, Brazil, Mexico, Peru, Uruguay, and the Caribbean islands. The species has perennial germination cycle, its leaves are prickly and, due to the beauty of its flowers, attracts pollinators like hummingbirds, increasing the natural dispersion of the species [186,187].

In folk medicine, the fruits of this species are used for respiratory problems such as flu, asthma, and bronchitis through the administration of homemade syrup produced by decoction. For the production of this syrup, the pulp of 1 *B. antiacantha* fruit is submitted to heating together with one cup of solvent, which in this case is water, for about 5 min; then the mixture is filtered and sugar is added in the proportion of two cups of sugar for one cup of the mixture. Sugar must be mixed under heating until complete homogenization and acts not only as sweetener, but also as a preservative, which is used according to the dose of one tablespoon three times a day [188,189].

In some cases, vegetable organs of other species are added in the syrup preparation to enhance the expectorant action. The most added species are *Achillea millefolium, Mentha sativa* and *Zingiber officinale*. Other indications for the use of the fruit are purgative, diuretic, vermifuge and abortion. Leaves are used in the form of tea prepared by infusion or decoction, with drops of propolis, used in mouthwash for the treatment of thrush and other disorders of the oral mucosa and the extract produced by maceration is indicated as antipyretic and anthelmintic [182,190,191].

#### 6.1.2. Botanical Aspects

*B. antiacantha* has stem with rhizomes about 1 m in length from which adventitious roots emerge. Rhizomes are covered by leaves and are responsible for the survival of the species under different climatic conditions. Leaves exhibit alternate and spiral phyllotaxis with 80 to 185 cm in length, arranged in non petiolated rosette without the cistern formation, with lanceolate and caniculate limb shape and aculeate limb margin [183,188].

Flowering is annual and occurs between the months of December and February. From the center of leaves, monopodial inflorescence emerges, which is composed of 150 to 350 meliophilous, ornithophilous and pedunculated flowers with oval sepals, entire margin of the sepal, oblong petal purple in color. During the flowering period, central leaves and bracts show intense red color. Fruits are polyspermic, fleshy, gaba type, yellow when ripe, with approximately 2 cm in diameter, pleasant odor, and edible pulp. Seeds are photoblastic, that is, they need sunlight to germinate, present 26% of moisture, with high germination rate at temperatures between 25 °C and 35 °C [78,183,191,192].

#### 6.1.3. Phytochemical Aspects

Table 4 presents a summary of classes of secondary metabolites found in the respective extracts and plant organs of *B. antiacantha* species.

There are few studies on the chemical composition of *B. antiacantha*, and most of them only perform qualitative screening, without quantifying or isolating substances. The analysis of the proximate composition of ripe fruits showed 82.63% of moisture, 0.62% of protein, 2% of fiber, 8.75% of carbohydrates and 0.93% of ash. In addition, other compounds were quantified, which are associated with the antioxidant activity of fruits and are composed of 70.73 mg/100 g of phenolic compounds, 162.67 mg/100 g of total carotenoids and 60.01 mg/100 g of vitamin C [197].

A qualitative phytochemical analysis performed with the methanolic extract from *B. antiacantha* leaves and fruits detected, in the methanolic extract of fruits, the following groups of secondary metabolites: alkaloids, phenols, flavonoids, tannins, triterpenes, steroids, anthraquinones and coumarins. In the methanolic extract of leaves, the following groups of secondary metabolites were identified: alkaloids, phenols, flavonoids, tannins, triterpenes, steroids and coumarins [193]. The presence of flavonoids, tannins and saponins was confirmed by analyses from another study, which also used the methanolic extract from *B. antiacantha* leaves and [182]. With regard to *B. antiacantha* fruits, the presence of hydroxycinnamic acids and flavone derivative was detected in the aqueous extract, which was not structurally identified [194]. The characterization of secondary metabolites of *B. antiacantha* species using the ethanolic extract to quantify flavonoids and the hydromethanolic extract to quantify anthocyanins showed: (1) in leaf extracts, 1.68 mg/mL of flavonoids and 0.0 mg/mL of anthocyanins and; (2) in bract extracts, 0.43 mg/mL of flavonoids and 10.83 mg/mL of anthocyanins. The greater production of anthocyanins in bracts may indicate the effort in the allocation of energy by plants to attract pollinators, since the amount of flavonoids found in leaves, although larger than in bracts, is still insignificant compared to other species that are sources of flavonoids [195]. Analyzing *B. antiacantha* leaves, [196] detected the presence of saponins in the methanolic extract and isolated saponin daucosterol (**63**), which was attributed the hemolytic property of the extract.

#### 6.1.4. Pharmacological Studies

The alcoholic extract from *B. antiacantha* fruits, and methanolic, hexanic, ethyl acetate and raw alcoholic extract from leaves were tested for antimicrobial, molluscicidal and antioxidant properties. All extracts were tested against clinically isolated *C. albicans* and *C. glabrata* strains and *C. albicans* (ATCC 90028), *E. coli* (ATCC 8739), *P. aeruginosa* (ATCC 9027) and *S. aureus* (ATCC 6538) reference strains. All strains tested were not affected by extracts, that is, none of the extracts showed antimicrobial or antifungal activity. The evaluated extracts were considered inactive in relation to the molluscicidal activity, since no significant effects were found at concentration of 400 μg/mL, a value above the maximum for this activity. To test the antioxidant activity, the performance of extracts against the DPPH radical was observed, showing unsatisfactory results, since only the extract from leaves with ethyl acetate obtained moderate performance, with 35% inhibition of radicals [182].

The methanolic (BAM), hexanic (BAH), dichloromethane (BAD), ethyl acetate (BAA) and hydromethanolic (BAHa) extracts from *B. antiacantha* leaves and fruits were submitted to antibacterial activity tests. Only BAM and BAD extracts from leaves and BAA extract from fruits inhibited the growth of *P. aeruginosa*, and the BAM extract from leaves showed activity on *E. coli* [193].

Daucosterol (**63**) is a saponin found in the methanolic extract from *B. antiacantha* leaves. This substance was the focus of a study that investigates its possible anti-inflammatory role on colitis induced by dextran sulfate sodium (DSS) in mice. Colitis is an inflammatory reaction in the large intestine, which source may be infectious or autoimmune. Pre or post-treatment with daucosterol (**63**) provided relief from the clinical symptoms of colitis, with reduction in the number of regulatory T cells, in the activity of Natural Killer (NK) cells and in the production of Immunoglobulin A (IgA), whose increase is characteristic of the disease. In addition, ROS inhibition and reduction in the expression of inflammatory cytokines such as TNF-α, IL-6, IL-1β and IFN-γ were observed, as well as increase in the anti-inflammatory cytokine IL-10 [198].

Induction of autophagic apoptosis in prostate cancer by daucosterol (**63**) was also analyzed, indicating anti-tumor activity. The action of this phytoconstituent on cancer cells promoted the interruption of the cell cycle by activating the mitochondrial-dependent apoptotic signaling pathway that leads to increased expression of the pro-apoptotic proteins caspase 3 and 9 and Bax, in addition to reducing the expression of Bcl-2. The administration of the 3-methyladenine (3-MA) autophagy inhibitor attenuated the apoptotic effect triggered by daucosterol (**63**), indicating that its mechanism of action is the induction of autophagic apoptosis. This mechanism may also be related to the action of JNK protein kinases, known for the regulatory role of cell proliferation, survival and death. Daucosterol (**63**) increased the level of JNK proteins active in cancer cells, while the specific JNK inhibitor (SP600125) inhibited its action, which indicates that this phytoconstituent has tumor suppressive effect through the induction of autophagic apoptosis dependent on the activation of the JNK signaling [199].

**Figure 7:**
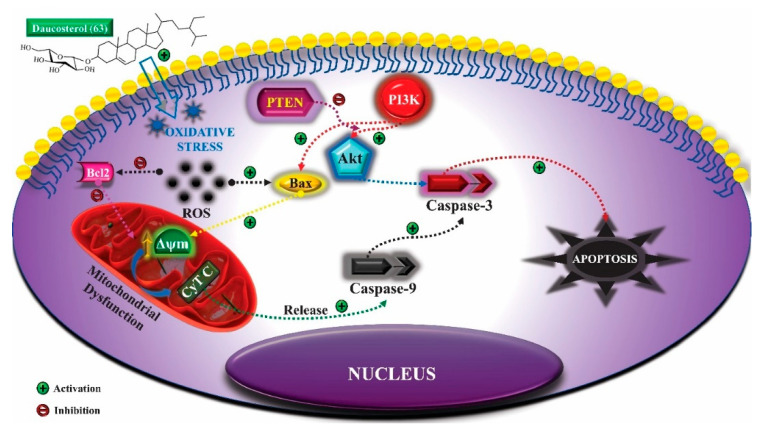
Daucosterol mechanism on human breast adenocarcinoma cells: After treatment of tumor cells (MCF-7) with daucusterol, a phytosterol abundantly present in *Bromelia antiacantha* extracts, the positive regulation of Phosphatase and Tensin Homologue (PTEN) blocks Protein Kinase B (Akt) activation through PI3K. Daucusterol induces reactive oxygen species (ROS) synthesis that leads to mitochondrial oxidative stress and, subsequently, release of cytochrome C. Subsequently, the activation of caspases causes cell apoptosis [202]. Δψm, mitochondrial membrane potential; Akt, Protein Kinase B; Bax, BCL2 Associated X; Bcl2, B-cell lymphoma 2; Cyt C, cytochrome C; PI3K, phosphatidylinositol-3-kinase; PTEN, phosphatase and tensin homologue; ROS, reactive oxygen species.

The apoptotic action promoted by daucosterol (**63**) has been investigated by several studies that correlate it with the anti-tumor action on breast [200] and prostate [201] cancers. Esmaeili et al. [202] investigated the anti-tumor mechanism of daucosterol (**63**) on human breast adenocarcinoma cells and concluded that the apoptotic mechanism is associated with the mitochondrial pathway, with loss of the mitochondria membrane potential and release of cytochrome C being observed after reduction of the Bcl-2/Bax ratio through the increase in the levels of intracellular ROS and decrease in the levels of antioxidant protein GSH and MMP. In addition, the PI3K/AKT pathway is inhibited by daucosterol (**63**) by reducing the AKT expression, whose levels are increased in some types of tumor. This reduction occurs by increasing the expression of the phosphatase and tensin homolog (PTEN), a negative regulator of the PI3K/AKT pathway. Thus, the inhibition of the PI3K/AKT pathway, the increase in Bax expression and the reduction of Bcl-2 levels, promote apoptosis mediated by the mitochondrial pathway and activation of caspases 3 and 9 (Figure 7) [202].

Another study also investigated the anti-tumor property of daucosterol (**63**), which inhibits the migration and invasion of hepatocellular carcinoma cells using another mechanism, the Wnt/β-catenin signaling pathway, which regulates various physiological processes such as cell proliferation, apoptosis, differentiation, transcription and translation. Daucosterol (**63**) acts by significantly inhibiting the expression of β-catenin, reducing the possibilities of cell proliferation, migration and invasion, which would occur through the Wnt/β-catenin pathway [203].

The neuroprotective effect of daucosterol (**63**) was also investigated in a study that reported the action of this compound as a modulator of the growth factor expression similar to insulin type 1 (IGF-1), which plays a neuroprotective role. Daucosterol (**63**) increased the level of AKT phosphorylation, resulting in more AKT in the active form and indicating that the AKT pathway was activated, resulting in protective effect on treated neurons, since the activation of the PI3K/AKT pathway favors cell survival, as it promotes the inactivation of GSK-3b, a pro-apoptotic protein, whose inhibition causes an increase in Mcl-1, which has the opposite effect, reducing the activity of caspase 3 [204].

#### 6.1.5. Toxicity Studies

Regarding the toxic effects of extracts obtained from *B. antiacantha*, a cytotoxicity test was carried out using *Artemia salina* nauplii, on which the alcoholic extracts from fruits and methanolic and alcoholic extracts from leaves of this plant showed toxicity, with LD_50_ values of 618.3 μg/mL, 275.9 μg/mL and 362.1 μg/mL, respectively [182]. In another study, hexane extracts in dichloromethane and ethyl acetate from *B. antiacantha* leaves showed toxicity against *Artemia salina*, with LD_50_ values of 53.9 μg/mL, 112.4 μg/mL and 241.6 μg/mL, respectively. For fruits, extract in dichloromethane was the only one with cytototoxic activity, with an LD_50_ of 29.8 μg/mL [193].

The hemolysis index promoted by the aqueous extract from *B. antiacantha* leaves and fruits was stipulated after analyzing lamb blood, observing total hemolysis in 0.85% and 1.00% dilutions for fruits, and 0.90% and 1.00% for leaves, while partial hemolysis occurred at 0.70% dilution for fruits and leaves. This hemolytic action was associated with the presence of saponin compounds in extracts, such as daucosterol (**63**), which were isolated from this species [196].

Saponins have cytotoxic activity, and this activity can occur through the promotion of autophagic cell death or cytoskeleton disintegration. In the case of hemolysis promoted by saponins, this may occur due to their ability to complex with cholesterol of the erythrocyte cell membrane, which results in the formation of pores in the membrane, increasing its permeability. In addition, some saponins, such as daucosterol (**63**) can act through signaling pathways such as PI3K/AKT, Wnt/β-catenin, promoting apoptosis. Autophagy induction can also occur by increasing the levels of light chain protein 3 (LC3) induced by some saponins. LC3 is associated with microtubules and is a marker for autophagy, being related to the formation of autophagic vacuoles [205,206].

## 7. Clinical Trials

Finally, it is essential to note that medicinal plants are used to prevent and treat diseases by humans, being used by about 80% of the population for primary health care. The rich biodiversity of the Brazilian cerrado offers a unique and incomparable potential for discovering and developing bioactive agents. Therefore, clinical trials with metabolites isolated from these species are of fundamental importance.

Once the pharmacological effect and the absence of side and toxic effects are proven, the test substance goes on to the clinical trial phase. Clinical trials involve research conducted on humans to discover or confirm the clinical and pharmacological effects observed during preclinical research. Besides, they identify adverse events and study the process of absorption, distribution, biotransformation, and secretion of the test substance. In the present study, we list the phytochemicals from the four reviewed plants that stood out during pre-clinical research and reviewed the clinical studies involving these compounds in the current literature. Table 5 summarizes these clinical studies.

All the species reviewed in the present study showed promising therapeutic potential due to the presence of phytochemicals. The species with the highest number of clinical trials found in the literature was *Talisia esculenta*. In general, clinical trials involving medicinal plants or their isolated secondary metabolites are scarce. However, studies like these are necessary for the development of more efficient pharmaceutical products for the treatment of various disorders that affect humans, in addition to being crucial for the health professional to be safe in prescribing these drugs.

## 8. Conclusions

The species under study reveal great economic importance not only in the consumption and marketing of fruits, but also as sources for the extraction of molecules with significant bioactive potential, which can be used as phytotherapeutic agents or as raw materials for the development of new drugs. *T. esculenta* has high concentration of phenolic acids, flavonoids, and terpenes, justifying its antioxidant, anti-tumor, anti-inflammatory and antimicrobial action reported by several studies, lacking studies that investigate toxicity associated with the ingestion of its seeds.

Future studies should perform quantitative analyses and isolation of substances from *B. gaudichaudii*, *G. americana* and *B. antiacantha*, favoring the understanding of the antiproliferative, antimicrobial, anti-inflammatory, neuroprotective, and photosensitizing effects associated with their extracts. Therefore, fruits of the Brazilian Cerrado offer an immeasurable richness of molecules with biological activity of great interest to the pharmaceutical and cosmetic industries, in addition to the possibility of marketing of fruits and their by-products. The knowledge of the mechanism of action of substances isolated in these extracts enables correlating concentration, effectiveness, desirable and side effects, which is fundamental for the establishment of a therapeutic planning and interventions in case of intoxication.

## Figures and Tables

**Table 1:** Phytochemical analysis of *T. esculenta*.

Compounds	Molecular Structures	Chromatographic Methods for the Isolation and Identification	Solvent Used/Essential Oil	Plant Part Used	Collection Site	References
Myricetin (**1**, F)	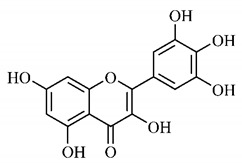	HPLC	Hydroalcoholic 5:95 (*v/v* water, ethanol)	Fruit	N.I.	[32]
Quercetin (**2,** F)	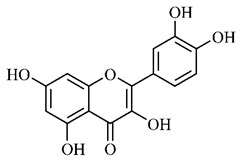					
Catechin (**3**, F)	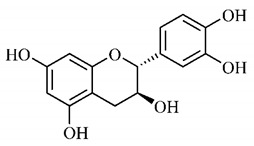	LC-MS	Acetone Methanol	Pulp Fruit peel Seed	Parintins, Amazonas-Brazil	[33]
Epicatechin (**4**, F)	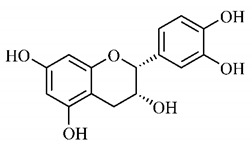		Acetone	Fruit peel Seed		
Gallic acid (**5**, PA)	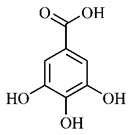		Acetone	Pulp		
Luteolin (**6**, F)	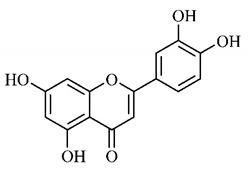		Acetone Methanol	Seed		
Naringenin (**7**, F)	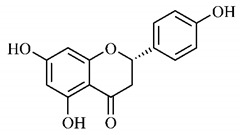		Acetone Methanol	Fruit peel Seed		
*p*-Coumaric acid (**8**, PA)	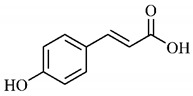		Acetone	Pulp		
Quinic acid (**9**, CL)	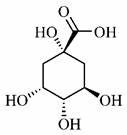		Acetone	Pulp		
Rutin (**10**, F)	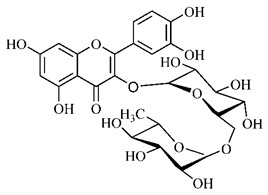		Acetone Methanol	Fruit peel		
Acacetin (**11**, F)	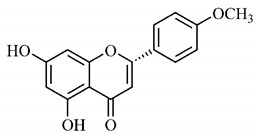	UHPLC–MS/MS	Methanol	Fruit	Manaus, Amazonas -Brazil	[34]
Caffeic acid (**12**, PA)	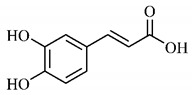					
Catechin (**3**, F)	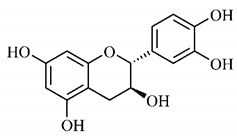					
Chlorogenic acid (**13**, PA)	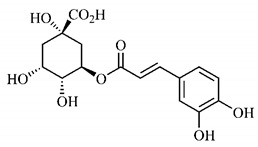					
Epicatechin (**4**, F)	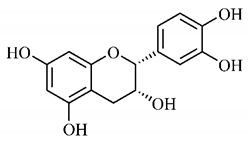					
Eriodictyol (**14**, F)	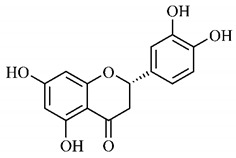					
Ferulic acid (**15**, PA)	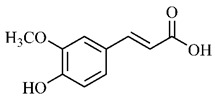					
Gallic acid (**5**, PA)	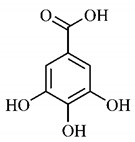					
*p*-Coumaric acid (**8**, PA)	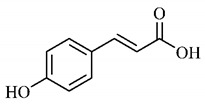					
Quercetin (**2**, F)	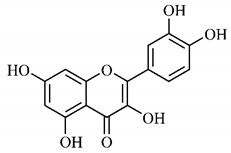					
Quinic acid (**9**, CL)	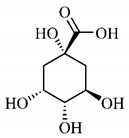					
Rutin (**10**, F)	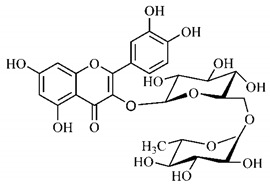					
Syringic acid (**16**, PA)	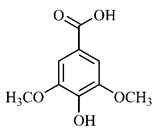					
β-Bisabolene (**17**, T)	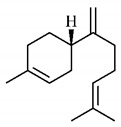	HS-SPME-GC-MS	DVB/CAR/PDMS			
Linalool (**18**, T)	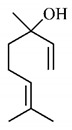					
Dihydroxybenzoic acid hexoside (**19**, PA)	*	UHPLC	Hydroalcoholic 1:1 (*v/v* methanol, water)	Leaf Stem	Dourados, Mato Grosso do Sul-Brazil	[35]
Kaempferol-diglycoside (**20**, F)	*					
Methylquercetin-diglycoside (**21**, F)	*					
Quercetin-diglycoside (**22**, F)	*					
Quercetin-rhamnoside (**23**, F)	*					
Dicaffeoylquinic acid (**24**, PA)	*					
Acacetin (**11**, F)	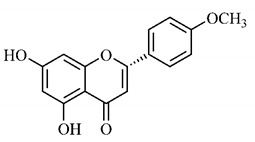	UHPLC–MS/MS	Hydroalcoholic 1:4 (*m/v*, 70% ethanol)	Leaf	São Luís, Maranhão-Brazil	[36]
Caffeic acid (**12**, PA)	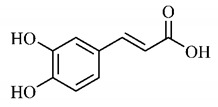					
Catechin (**3**, F)	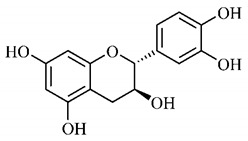					
Gallic acid (**5**, PA)	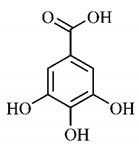					
Quercetin (**2**, F)	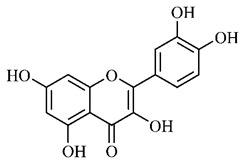					
Quinic acid (**9,** CL)	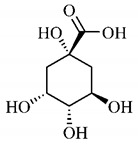					
Rutin (**10**, F)	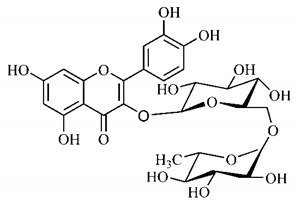					

N.I.: not identified; CL: cyclitol; F: flavonoid; PA: phenolic acid; T: terpenes; HPLC: High Performance Liquid Chromatography; HS-SPME-GC-MS: Headspace Solid Phase Microextraction/Gas Chromatography with Mass Spectrometry Detection; LC-MS: Liquid Chromatography Coupled to Mass Spectrometry; UHPLC: Ultra-High Performance Liquid Chromatography; UHPLC–MS/MS: Ultra-High Performance Liquid Chromatography associated with Mass Spectrometry; DVB/CAR/PDMS: Divinylbenzene/Carboxen/Polydimethylsiloxane. * Based on the lack of specificity in the indication of the substitution chemical groups it was not possible to design the structures once the article states that it was a tentative identification

**Table 2:** Phytochemical analysis of *B. gaudichaudii*.

Compounds	Molecular Structures	Chromatographic Methods for the Isolation and Identification	Solvent Used/Essential Oil	Plant Part Used	Collection Site	References
Psoralen (**25**, C)	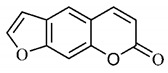	HPLC	Methanol	Root cortex Leaf Twigs Latex Heartwood of the root Root cortex	São Paulo-Brazil	[91]
Bergapten (**26**, C)	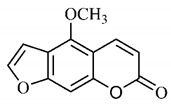					
5,7,3‘,4’-Tetrahydroxy-6-*C*-glucopyranosylflavone (**27**, F)	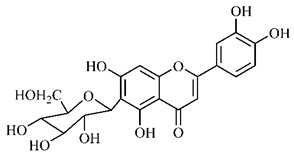		Water	Root cortex		
28 4 5,7,3‘,4’-tetrahydroxy-3-*O*-β-d-galactopyranosylflavonol (F)	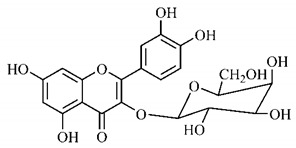					
Psoralen (**25**, C)	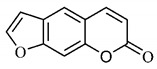					
Bergapten (**26**, C)	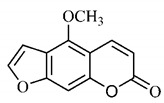					
Psoralen (**25**, C)	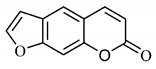	HPLC	Ethanol (*v/v* 95%)	Root	Jussára, Goiás-Brazil	[92]
Bergapten (**26**, C)	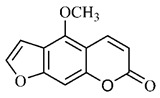					
Gaudichaudine (**29**, C)	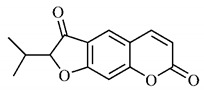	HPLC	Dichloromethane	Root bark	Araguari, Minas Gerais-Brazil	[93]
Luvangetin (**30**, C)	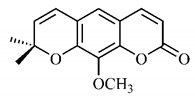					
(+)-(2’*S*,3’*R*)-3′-hydroxymarmesin (**31**, C)	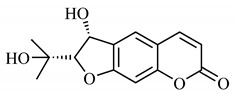					
Xanthyletin (**32**, C)	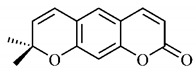					
Psoralen (**25**, C)	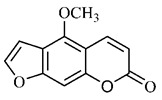					
Bergapten (**26**, C)	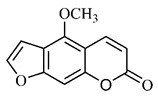					
Marmesin (**33**, C)	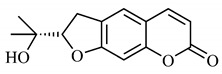	CC	Dichloromethane	Root bark	Araguari, Minas Gerais-Brazil	[94]
1′,2′-Dehydromarmesin (**34**, C)	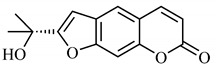					
8-Methoxymarmesin (**35**, C)	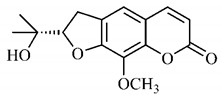					
1′-Hydroxy-3′-*O*-β-glucopyranosylmarmesin (**36**, C)	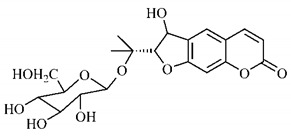					
2′,4′,4-Trihydroxy-3′3-diprenylchalcone (**37**, CH)	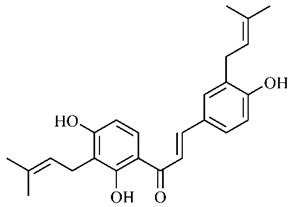					
β-Amyrin (**38**, T)	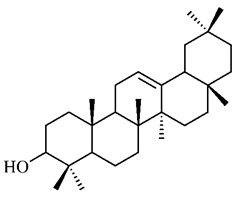					

C: coumarin; CH: chalcone; F: flavonoid; T: terpene; CC: Column Chromatography; HPLC: High Performance Liquid Chromatography.

**Table 3:** Phytochemical analysis of *G. americana.*

Compounds	Molecular Structures	Chromatographic Methods for the Isolation and Identification	Solvent Used/Essential Oil	Plant Part Used	Collection Site	References
Asystasioside D (**39**, T)	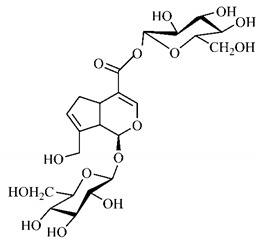	UHPLC	Hydroalcoholic 70:30 (*v/v* ethanol, water)	Leaf	Natal, Rio Grande do Norte-Brazil	[139]
Geniposidic acid (**40**, T)	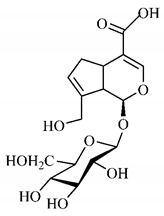					
Tarenoside (**41**, T)	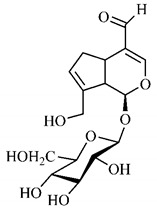					
Teneoside A (**42**, T)	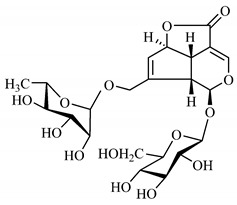					
Kaempferol-3-*O*-hexoside-deoxyhexoside-7-*O*-deoxyhexoside (**43,** F)	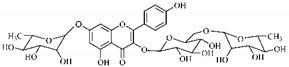					
Isorhamnetin-3-*O*-hexoside-deoxyhexoside-7-*O*-deoxyhexoside (**44**, F)	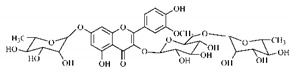					
Quercetin-3-*O*-hexoside-deoxyhexoside (**45**, F)	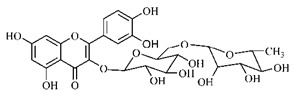					
Kaempferol-3-*O*-hexoside-deoxyhexoside (**46**, F)	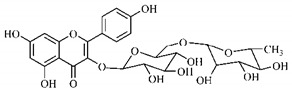					
Isorhamnetin-3-*O*-hexoside-deoxyhexoside (**47**, F)	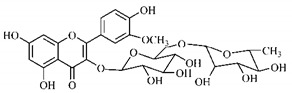					
Geniposidic acid (**40**, T)	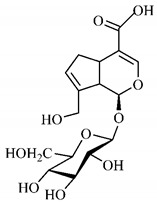					[140]
Geniposide (**48**, T)	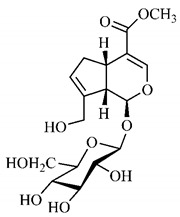					
Genipin-gentiobioside (**49**, T)	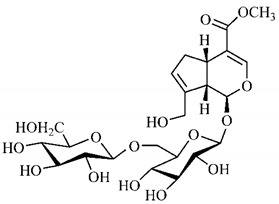					
Genameside A (**50**, T)	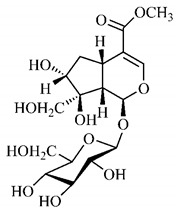					
Genameside B (**51**, T)	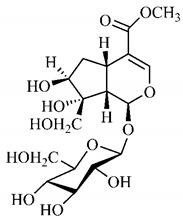					
Genameside C (**52**, T)	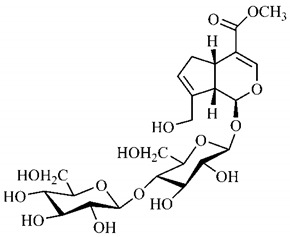					
Genameside D (**53,** T)	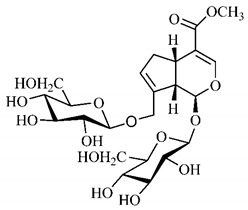					
Gardenoside (**54**, T)	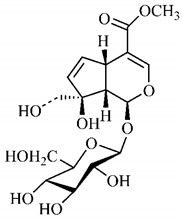					
Genipin (**55**, T)	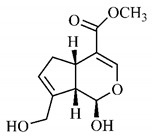	HPLC	Hydroalcoholic (*v/v*, 80% methanol, ultra-pure water)	Fruit	São Paulo-Brazil	[141]
1-Hydroxy-7-(hydroxymethyl)-1,4*aH*,5*H*,7a*H*-cyclopenta[*c*]pyran-4-carbaldehyde (**56**, T)	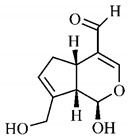	HPLC	Hydroalcoholic (*v/v*, 70 % ethanol, water)	Leaf	Natal, Rio Grande do Norte-Brazil	[142]
7-(Hydroxymethyl)-1-methoxy-1*H*,4*aH*,5*H*,7*aH*-cyclopenta[*c*]pyran-4-carbaldehyde (**57**, T)	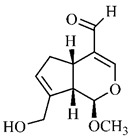					
Geniposidic acid (**40**, T)	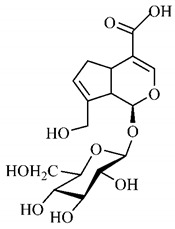	UHPLC	Methanol	Mesocarp Endocarp	Campinas, São Paulo-Brazil	[143]
Gardenosid (**54**, T)	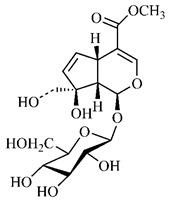					
Genipin-1-β-gentiobioside (**49**, T)	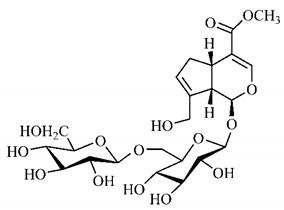					
Geniposide (**48**, T)	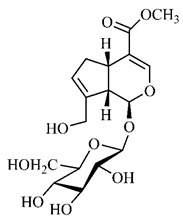					
6′’-*O*-*p*-Coumaroyl-1-β-gentiobioside geniposidic acid (**59**, T)	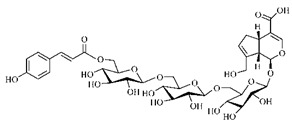					
6′′-*O*-*p*-Coumaroylgenipin-gentiobioside (**60**, T)	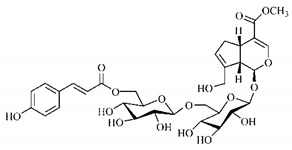					
Genipin (**55**, T)	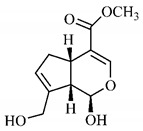					
6′-*O*-*p*-Coumaroyl-geniposidic acid (**61**, T)	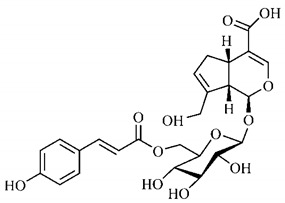					
6′-*O*-Feruloyl-geniposidic acid (**62**, T)	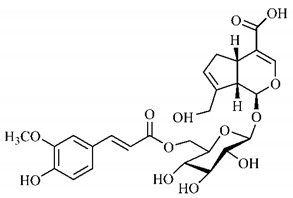					
Genipin (**55**, T)	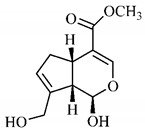	HPLC	Ethanol	Mesocarp Seeds Fruit peel Whole fruit Endocarp	Paraibuna, São Paulo-Brazil	[144]
Geniposide (**48**, T)	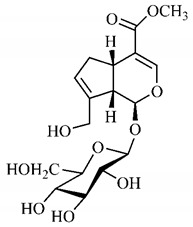					

F: flavonoid; T: terpene; HPLC: High Performance Liquid Chromatography; UHPLC: Ultra-High Performance Liquid Chromatography.5.1.4. Pharmacological Studies.

**Table 4:** Phytochemical analysis of *B. antiacantha.*

Compounds	Molecular Structures	Chromatographic Methods for the Isolation and Identification	Solvent Used/Essential Oil	Plant Part Used	Collection Site	References
Alkaloid	*	_	Methanol	Fruit	Rio Pomba, Minas Gerais-Brazil	[193]
Flavonoid	*					
Tannin	*					
Terpene	*					
Anthraquinone	*					
Coumarin	*					
Alkaloid	*			Leaf		
Flavonoid	*					
Tannin	*					
Terpene	*					
Coumarin	*					
Flavonoid	*	_	Methanol	Fruit Leaf	Umuarama, Paraná-Brazil	[182]
Tannin	*					
Saponin	*					
Flavonoid	*	_	Water	Fruit	Vale do Itajaí, Santa Catarina-Brazil	[194]
Flavonoid	*	_	Hydroalcoholic	Leaf	Viamão, Rio Grande do Sul-Brazil	[195]
Anthocyanin	*					
Flavonoid	*			Bract		
Anthocyanin	*					
Daucosterol (**63**, S)	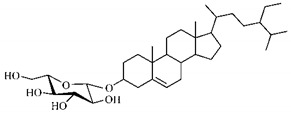	CC	Methanol	Leaf	Umuarama, Paraná-Brazil	[196]

S: saponin; CC: Column Chromatography. * Based on the lack of the isolation study of the chemical constituent, as it is a qualitative study, it was not possible to design the structures.

**Table 5:** Clinical trials with secondary metabolites of fruit plants belonging to the Brazilian cerrado.

Species	Compounds	Investigated Pathology	Search Result	References
*Talisia esculenta*	Catechin (**3**)	Hypercholesterolemia	Hypolipidemic and hepatoprotective effect	[207]
		Obesity and type 2 diabetes	Reduction of visceral fat, blood pressure and cholesterol	[208]
		Coronary artery disease	Reduction of oxidized LDL in plasma	[209]
		Child obesity	Reduction in waist circumference, systolic blood pressure and LDL levels	[210]
	Epicatechin (**4**)	Vascular dysfunction	Improved cardiovascular health	[211]
		Arterial hypertension	Ineffective on blood pressure, blood lipid profile and glucose control	[212]
		Cardiovascular diseases	Cardioprotective effect and improved insulin resistance	[213]
	Quercetin (**2**)	Arterial hypertension	Lowering blood pressure	[214]
		Increase in free radicals produced after eccentric exercises	Antioxidant and protective effect	[215]
		Hyperuricemia	Significant reduction in elevated plasma uric acid concentrations	[216]
		Rheumatoid arthritis	Significant improvement in clinical symptoms and reduced levels of TNF-α	[217]
		Gout and primary hypertension	Improvement of echocardiographic parameters, left ventricular diastolic function, purine metabolism, renal function, and normalization of blood pressure	[218]
		Overweight or obesity with polycystic ovary syndrome	Significant reduction in gene expression and plasma resistin concentration and considerable decrease in the level of luteinizing hormone and testosterone	[219]
		β-Thalassemia major	Reduced iron overload	[220]
	Chlorogenic acid (**13**)	Polycystic ovary syndrome	Improvement of insulin resistance and hormonal profile of women with the syndrome.	[221]
		Neuromuscular dysfunction	Improvement of neuromuscular performance	[222]
		Dyslipidemia	Decrease in cholesterol, triglycerides and LDL values, and increased HDL levels	[223]
		Sarcoidosis	Reduction of oxidative stress and inflammation	[224]
		Hypertension and fat accumulation	Reduction of blood pressure and body fat	[225]
		Cognitive dysfunction	Improvement of cognitive functions	[226]
		Arterial hypertension	Reduction of systolic and diastolic blood pressure	[227]
*Brosimum gaudichaudii*	Psoralen (**25**)	Fungal ringworm	Oral treatment with a low dose and low frequency of psoralen-UV-A, was safe and effective	[228]
		Chronic palmar eczema of the hand	Reduced severity of chronic palmar eczema of the hand	[229]
		Chronic moderate to severe plaque psoriasis	Psoralen plus ultraviolet A, are therapeutic options for chronic moderate to severe plaque psoriasis	[230]
		Cutaneous mastocytosis	Efficacy for the treatment of moderate to severe chronic psoriasis	[231]
		Vitiligo	Increased extent of skin repigmentation	[232]
	Bergaptene (**26**)	Psoriasis	Reduction of signs and symptoms of psoriasis	[233]
*Genipa americana*	Genipin (**55**)	Absence of clinical trials in the current literature	_	_
	Asystasioside D (**39**)			
	Geniposidic acid (**40**)			
	Tarenoside (**41**)			
*Bromelia antiacantha*	Daucosterol (**63**)	Anogenital warts	The treatment led to the elimination of injury	[234]
		Pulmonary Tuberculosis	Improvement in imaging tests and weight gain of the patient	[235]
